# A family of *C*. *elegans* VASA homologs control Argonaute pathway specificity and promote transgenerational silencing

**DOI:** 10.1016/j.celrep.2022.111265

**Published:** 2022-09-06

**Authors:** Siyuan Dai, Xiaoyin Tang, Lili Li, Takao Ishidate, Ahmet R. Ozturk, Hao Chen, Altair L. Dude, Yong-Hong Yan, Meng-Qiu Dong, En-Zhi Shen, Craig C. Mello

**Affiliations:** 1 RNA Therapeutic Institute, UMass Chan Medical School, Worcester, MA 01605, USA; 2 Morningside Graduate School of Biomedical Sciences, UMass Chan Medical School, Worcester, MA 01605, USA; 3 Key Laboratory of Growth Regulation and Translational Research of Zhejiang Province, School of Life Sciences, Westlake University, Hangzhou, Zhejiang, China; 4 Westlake Laboratory of Life Sciences and Biomedicine, Hangzhou, Zhejiang, China; 5 National Institute of Biological Sciences, Beijing 102206, China; 6 Howard Hughes Medical Institute, Worcester, MA 01605, USA; 7 Program in Bioinformatics and Integrative Biology, UMass Chan Medical School, Worcester, MA 01605, USA; 8 Bioinformatics Program, Boston University, Boston, MA 02215, USA; 9 These authors contributed equally; 10 Lead contact

## Abstract

Germline Argonautes direct transcriptome surveillance within perinuclear membraneless organelles called nuage. In *C*. *elegans*, a family of Vasa-related Germ Line Helicase (GLH) proteins localize in and promote the formation of nuage. Previous studies have implicated GLH proteins in inherited silencing, but direct roles in small-RNA production, Argonaute binding, or mRNA targeting have not been identified. Here we show that GLH proteins compete with each other to control Argonaute pathway specificity, bind directly to Argonaute target mRNAs, and promote the amplification of small RNAs required for transgenerational inheritance. We show that the ATPase cycle of GLH-1 regulates direct binding to the Argonaute WAGO-1, which engages amplified small RNAs. Our findings support a dynamic and direct role for GLH proteins in inherited silencing beyond their role as structural components of nuage.

## In brief

Dai et al. show that worm VASA homologs, GLH proteins, long known as factors that promote perinuclear nuage formation in germ cells, are in fact direct participants in Argonaute surveillance. GLH proteins directly bind mRNAs targeted by Argonautes and utilize their ATPase domains to regulate both mRNA and Argonaute binding.

## Graphical Abstract

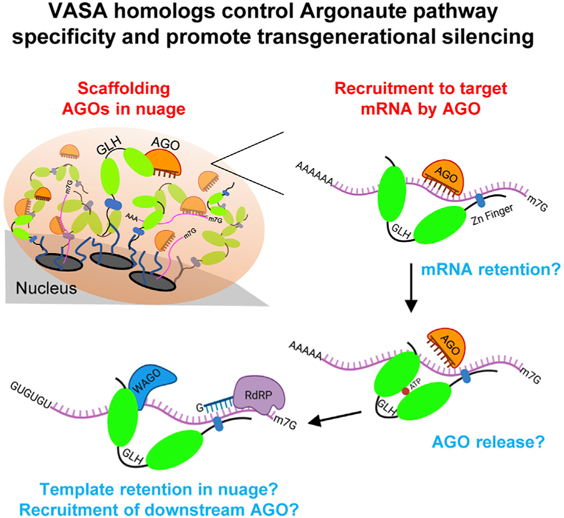

## INTRODUCTION

In diverse animals, germline Argonautes direct the transgenerational silencing of transposons and many developmentally important genes (Aravin et al., 2007; Klattenhoff and Theurkauf, 2008; Ozata et al., 2019). In *C*. *elegans* anti-sense small RNAs (22G-RNAs) produced by cellular RNA-dependent RNA polymerases (RdRPs) engage WAGO and CSR-1 Argonautes to target nearly all germline-expressed mRNAs. Both RdRP templating and Argonaute surveillance are thought to occur within perinuclear liquid-like condensates called nuage or P granules (Brangwynne et al., 2009; Pitt et al., 2000; Strome and Wood, 1982, 1983; Wolf et al., 1983). Whereas WAGOs mediate silencing, CSR-1 both modulates gene expression (Gerson-Gurwitz et al., 2016) and protects from silencing (Avgousti et al., 2012; Claycomb et al., 2009; de Albuquerque et al., 2015; Gu et al., 2009; Seth et al., 2013; Tu et al., 2015). The piRNA/PRG-1 pathway engages all transcripts, providing a scanning function that relies on microRNA-like base pairing (Shen et al., 2018). PRG-1 recruits RdRP to produce WAGO-associated 22G-RNAs adjacent to PRG-1/Piwi-interacting RNA (piRNA) binding sites on many germline mRNAs, and also promotes small-RNA amplification upon encountering foreign RNA sequences (not targeted by CSR-1) (Ashe et al., 2012; Lee et al., 2012; Shirayama et al., 2012).

Amplification of WAGO 22G-RNAs and transgenerational silencing can be stimulated by double-stranded RNA (dsRNA) through the canonical RNAi pathway, which employs a distinct upstream Argonaute, RDE-1 (Gu et al., 2009; Pak and Fire, 2007). WAGO 22G-RNAs, in turn, direct transgenerational gene silencing (Ashe et al., 2012; Buckley et al., 2012; Lee et al., 2012; Shirayama et al., 2012). The canonical RNAi pathway and the PRG-1/piRNA pathway compete with each other for the WAGO amplification and inheritance machinery (Luteijn et al., 2012; Shukla et al., 2021). Although PRG-1/piRNA complexes engage CSR-1 targets, these interactions—though energetically favorable—fail to stimulate WAGO 22G-RNA accumulation in most cases (Shen et al., 2018).

DEAD-box proteins have been studied extensively and are known to regulate RNA-RNA and RNA-protein interactions (Linder and Fuller-Pace, 2015). Dead-box proteins are also known to function in a number of Argonaute small-RNA pathways (Pek and Kai, 2011; Wenda et al., 2017; Xiol et al., 2014; Zhang et al., 2012). In insects, for example, VASA functions within nuage to promote the amplification of piRNAs required to suppress transposons (Xiol et al., 2014). In *C*. *elegans*, the DEAD-box protein RDE-12 functions with the Argonautes RDE-1 and WAGO-1 to promote 22G-RNA production during RNAi (Shirayama et al., 2014; Yang et al., 2014).

Several studies have uncovered roles for a family of Germ Line DEAD-box Helicase (GLH) family proteins in the regulation of germline RNAs (Beshore et al., 2011; Dallaire et al., 2018; Gruidl et al., 1996). GLH-1 has also been implicated in transgenerational inheritance of RNAi (Spracklin et al., 2017). Worms with loss-of-function mutations in *glh-1* are viable at 20°C but become sterile after multiple generations at 25°C, known as a mortal germline phenotype (Kuznicki et al., 2000; Spike et al., 2008a; Spracklin et al., 2017). *glh-4*-null mutant worms exhibit modest fertility defects, but *glh-4 glh-1* double mutants are sterile at all temperatures (Kuznicki et al., 2000; Spike et al., 2008a). Mutations in *glh-1* perturb the localization of other P-granule components, including PGL-1 (Chen et al., 2020; Kuznicki et al., 2000; Marnik et al., 2019; Spike et al., 2008a). Several P-granule components become completely dispersed in *glh-4 glh-1* double mutants (Chen et al., 2020; Spike et al., 2008a). GLH proteins have intrinsically disordered regions (IDRs), which contain phenylalanine-glycine (FG) repeats that promote P-granule association with nuclear pores (Updike et al., 2011; Marnik et al., 2019). In addition to IDR and helicase domains, GLH proteins contain several copies of a retroviral-type (CCHC) zinc-finger domain that is also found in the RNA-binding protein LIN-28 (Moss et al., 1997; Wilbert et al., 2012).

Here, we show that GLH-1 and GLH-4 interact with the Argonautes PRG-1 and WAGO-1, as previously reported (Chen et al., 2020; Marnik et al., 2019). We show that GLH-1 and GLH-4 preferentially bind WAGO target mRNAs as measured by crosslinking and immunoprecipitation (CLIP), and that binding is diminished in mutants that compromise PRG-1-dependent silencing. We show that GLH-1(K391A), a lesion predicted to disrupt ATP binding by the helicase domain, prevents RNA-duplex unwinding *in vitro* but does not prevent GLH-1 RNA binding as measured by both *in vivo* CLIP and *in vitro* gel-shift assays. Rather, GLH-1(K391A) binding is enhanced on WAGO target RNAs *in vivo*. A GLH-1 DQAD lesion, predicted to prevent ATP hydrolysis, causes GLH-1 to bind RNA more strongly *in vitro*. However, interestingly, GLH-1(DQAD) loses its preference for WAGO target binding *in vivo* and instead binds many abundant mRNAs including CSR-1 targets.

Our findings suggest that, along with its paralogs, GLH-1 promotes and regulates Argonaute-mediated mRNA surveillance in at least three ways. First, special alleles and double mutants that disrupt P granules also disrupt small-RNA levels in the three major nuage-associated Argonaute pathways—i.e., WAGO, CSR-1, and PRG-1—suggesting that GLH proteins help establish a scaffold for Argonaute-mediated surveillance. Second, null alleles of *glh-1* cause a loss of 22G-RNAs on WAGO targets and ectopic 22G-RNAs on many other target mRNAs. The ectopic 22G-RNAs depend on GLH paralogs and PRG-1 activity, suggesting that GLH-1 prevents other GLH paralogs from inducing piRNA-initiated overproduction of 22G-RNA on many targets. Third, we show that GLH-1(K391A) strongly impairs transgenerational silencing and WAGO small-RNA production without disrupting P granules or other Argonaute small-RNA levels. GLH-1(K391A) exhibits a marked increase in its association with the WAGO-1 protein both *in vitro* and *in vivo*, suggesting that when unable to bind ATP, GLH-1 prevents WAGO-1 or co-factors from engaging GLH paralogs, but does so without disrupting GLH protein scaffolding functions required for other Argonaute pathways in nuage. Taken together, our work supports a model in which GLH proteins are recruited by Argonautes to bind and retain target mRNAs within nuage-promoting pathway-specific transgenerational inheritance of small-RNA signals.

## RESULTS

### GLH-1 and GLH-4 promote piRNA-induced silencing

**Figure 1.**
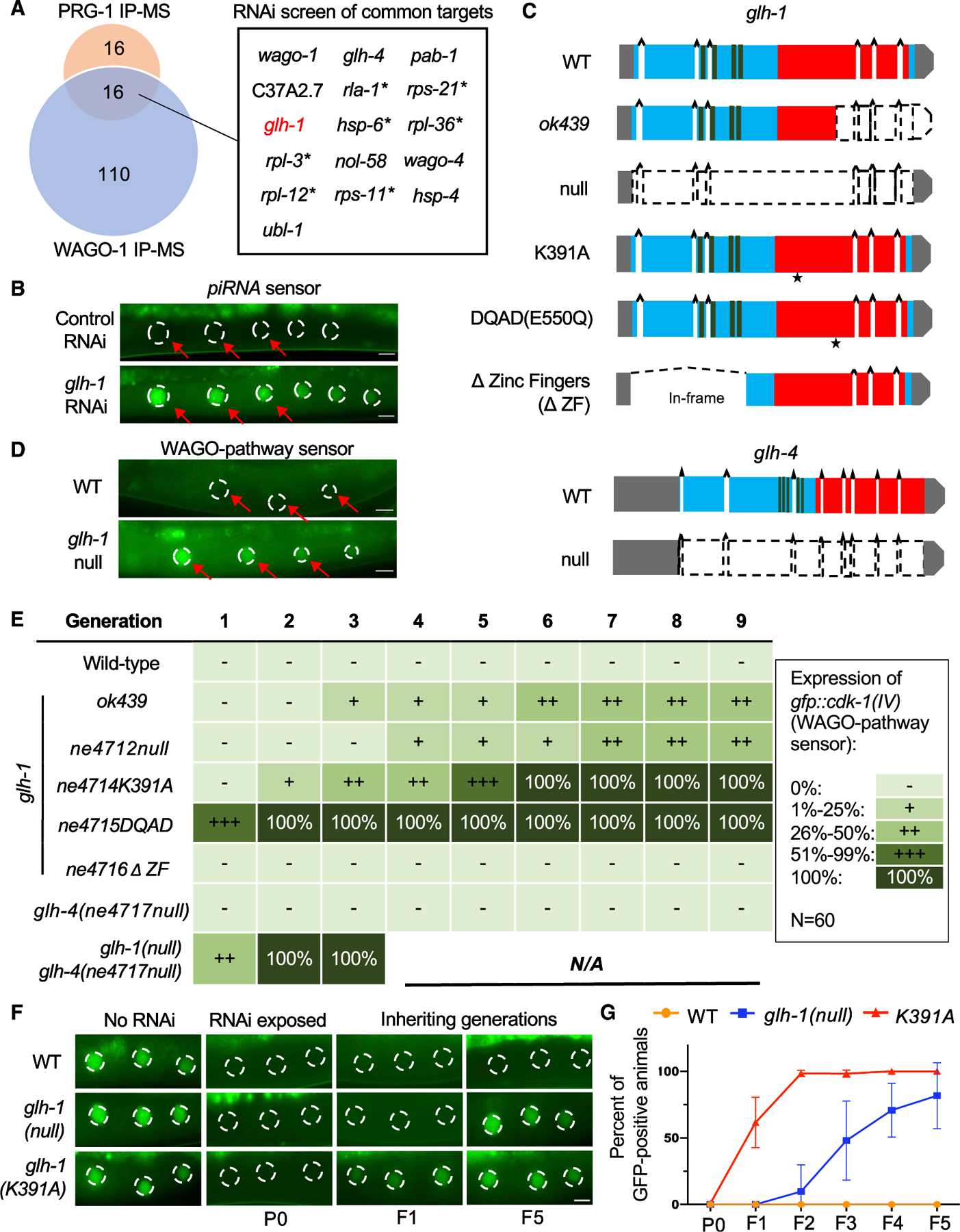
GLH-1 and GLH-4 promote piRNA-induced silencing and inherited RNAi (A) PRG-1 and WAGO-1 IP-MS identified an overlapping set of 16 high-confidence interactors. RNAi-mediated knockdown of these interactors in piRNA pathway sensors identified GLH-1 as required for both initiation and maintenance of piRNA-mediated silencing (see STAR Methods). Asterisks indicate that RNAi arrests larval development, preventing further analysis. The ribosomal proteins recovered in our IPs strongly overlap with those recovered by FLAG:GFP IP (Marnik et al., 2019), and therefore could be background. (B) Representative fluorescence images showing germline silencing or activation of a piRNA sensor *cdk-1*::*gfp*(IV); *21ux anti-gfp* piRNA(X) in oocyte nuclei of worms treated with control RNAi (L4440) or *glh-1* RNAi. Oocyte nuclei are outlined by dashed lines and indicated with red arrowheads. Scale bars, 10 μm. (C) Schematic diagram depicting *glh-1* and *glh-4* gene structures, locations of annotated domains, and mutations analyzed in the study. Asterisks indicate amino acid substitution sites. (D) Representative fluorescence images showing germline silencing or activation of a WAGO-pathway sensor *gfp*::*cdk-1*(IV) in wild-type (WT) or *glh-1-null* worms. Scale bars, 10 μm. (E) Penetrance of WAGO-pathway sensor expression in wild-type and glh mutant worms over nine generations. Five categories of penetrance were established depending on the percentage of animals expressing the sensor: –, 0%; +, 1%–25%; ++, 26%–50%; +++, 51%–99%; and 100%. At least 60 animals were scored at each generation. (F) Representative images from RNAi inheritance assays showing silencing or expression of a *cdk-1*::*gfp* reporter in wild-type (WT), *glh-1-*null, or *glh-1*(*K391A*) worms grown on control plates (no RNAi) or on *gfp* RNAi plates in the P0 generation, and then in the first and fifth generations after removal from *gfp* RNAi. Scale bar, 10 μm. (G) Graph of RNAi inheritance assays from (F) plotting the percentage of WT, *glh-1-*null, or *glh-1*(*K391A*) worms expressing *cdk-1*::*gfp* at each generation. Worms were exposed to *gfp* RNAi (P0) and removed from *gfp* RNAi for subsequent generations (F1 to F5). Error bars indicate standard deviation from the mean.

To identify factors that promote the recruitment of the WAGO silencing machinery by the piRNA pathway, we purified proteins that associate with both the Piwi Argonaute PRG-1 and with the prominent P-granule-localized Argonaute WAGO-1. We used CRISPR to engineer worms expressing FLAG-tagged PRG-1 or WAGO-1 from the endogenous *prg-1* and *wago-1* genes. We then used FLAG-specific antibodies to recover co-precipitated factors followed either by liquid chromatography-tandem mass spectrometry (LC-MS/MS), or by denaturing or native polyacrylamide gel electrophoresis (PAGE) to separate bands containing prominent co-factors for immunoprecipitation mass spectrometry (IP-MS) (Figure S1A). Using these approaches, we identified 32 proteins enriched by FLAG:PRG-1 IP (Figure 1A and Table S1A). A similar analysis of the FLAG:WAGO-1 IP identified 126 proteins, including 16 proteins also enriched in the PRG-1 IP (Figure 1A and Table S1B).

Because we wished to find factors that bridge initial targeting by PRG-1 to inheritance maintained by WAGO Argonautes, we asked if candidates pulled down by both WAGO-1 and PRG-1 are required for silencing of two different piRNA pathway reporters: a *cdk-1*::*gfp* reporter, whose silencing depends on an artificial anti-*gfp* piRNA (Shen et al., 2018) (Figure 1B); and a *gfp*::*cdk-1* reporter, whose silencing is initiated by PRG-1, but maintained by the WAGO pathway independently of PRG-1 activity (Shirayama et al., 2012). RNAi-mediated knockdown of *glh-1* desilenced both reporters, suggesting that GLH-1 bridges initial targeting by PRG-1 and maintenance of silencing by WAGOs.

Previous studies found that GLH-1 and GLH-4 function together (Kuznicki et al., 2000; Spike et al., 2008a). To investigate whether and how GLH-1 and GLH-4 promote piRNA silencing, we generated a series of *glh* mutant alleles that result in complete removal of *glh-1* or *glh-4* coding regions (null), in-frame deletion of the zinc-finger domains from GLH-1 (∆ZF), or specific amino acid substitutions in conserved residues of GLH-1 predicted to prevent ATP binding (K391A) or ATP hydrolysis (E550Q; DEAD to DQAD) (Figure 1C). Mutations that disrupt ATP binding prevent or reduce RNA binding by DEAD-box proteins (Cheng et al., 2005; Sengoku et al., 2006; Xiol et al., 2014), whereas mutations that disrupt ATP hydrolysis block the release of bound RNA (Hondele et al., 2019; Pause and Sonenberg, 1992; Xiol et al., 2014).

Complete deletion of *glh-1* did not initially desilence the piRNA sensor or the WAGO-pathway reporter, but both reporters were expressed in a fraction of *glh-1-*null worms by the fourth homozygous generation, and the percentage of expressing animals increased in subsequent generations (Figures 1D and 1E). Interestingly, the K391A and DQAD lesions caused stronger desilencing phenotypes than did the *glh-1*-null alleles (Figure 1E). For example, the WAGO-dependent but PRG-1-independent *gfp*::*cdk-1* reporter was desilenced in 31% of the K391A mutant worms in the second homozygous generation and in 93% of DQAD homozygotes in the first generation. By contrast, in-frame deletion of the CCHC zinc-finger domains in *glh-1*(*∆ZF*) worms had no effect on silencing (Figure 1E). The onset and extent of silencing defects in these *glh-1* mutants was mirrored by deficits in fertility and embryonic viability (Figures S1B and S1C).

The fact that GLH-1 K391A and DQAD point mutants show more severe phenotypes than null mutants suggests that the mutant K391A and DQAD proteins might interfere with the function of paralogs—e.g., GLH-4—which could otherwise partially compensate for the loss of GLH-1 (Spike et al., 2008a). Indeed, although both reporters remained silent in *glh-4-*null worms through nine generations, they were desilenced in *glh-4-*null *glh-1-*null double-mutant worms in the first generation (Figure 1E), a desilencing phenotype similar to that observed for *glh-1*(*DQAD*) mutants. These results show that GLH-1 and GLH-4 function redundantly in small-RNA silencing.

### GLH proteins function in inherited RNAi and prevent cold-sensitive defects in RNAi initiation

Previous studies have shown that *glh-1-*null mutants have an intact RNAi response in animals exposed to dsRNA, but exhibit reduced transmission of silencing to unexposed progeny, an RNAi inheritance defect (Spracklin et al., 2017). Similarly, we found that *glh-1-*null mutants exhibited complete silencing of a *cdk-1*::*gfp* reporter when *gfp* dsRNA was present, but failed to maintain silencing in inheriting generations after removal from dsRNA. For example, whereas wild-type animals transmitted silencing to all progeny in each of the first five generations after dsRNA removal (0% recovery), *glh-1-*null animals gradually recovered (Figures 1F and 1G). GLH-1(K391A) animals were also fully sensitive to dsRNA silencing in exposed animals but were more defective than the null in RNAi inheritance, reaching nearly 100% desilencing by the second generation (Figure 1G).

We were surprised to find that presumptive null (deletion or degron) alleles of *glh-1* and *glh-4*—though robustly sensitive to RNAi at room temperature—were partially resistant to RNAi at lower temperatures, a cold-sensitive RNAi (csRNAi) defect (Figures S1D–S1F). Notably, despite its usually stronger phenotypes, *glh-1*(*K391A*) worms did not show a csRNAi defect, but instead remained 100% sensitive to RNAi at all temperatures (Figure S1D). Since *glh*-null alleles caused ectopic PRG-1-dependent 22G-RNAs (see below), but *glh-1*(*K391A*) did not, we wondered whether misdirection of the PRG-1 pathway might cause the csRNAi defects. Consistent with this idea, a *prg-1* loss-of-function mutation completely suppressed the csRNAi defects of *glh-1* and *glh-4*-null mutants (Figures S1E and S1F). Taken together, these findings suggest that the GLH proteins promote inheritance of both dsRNA- and piRNA-induced silencing and also function to prevent a misdirection of the piRNA pathway that causes a cold-sensitive deficit in the dsRNA response (see discussion).

### GLH-1 lesions affect the localization of P-granule components

**Figure 2.**
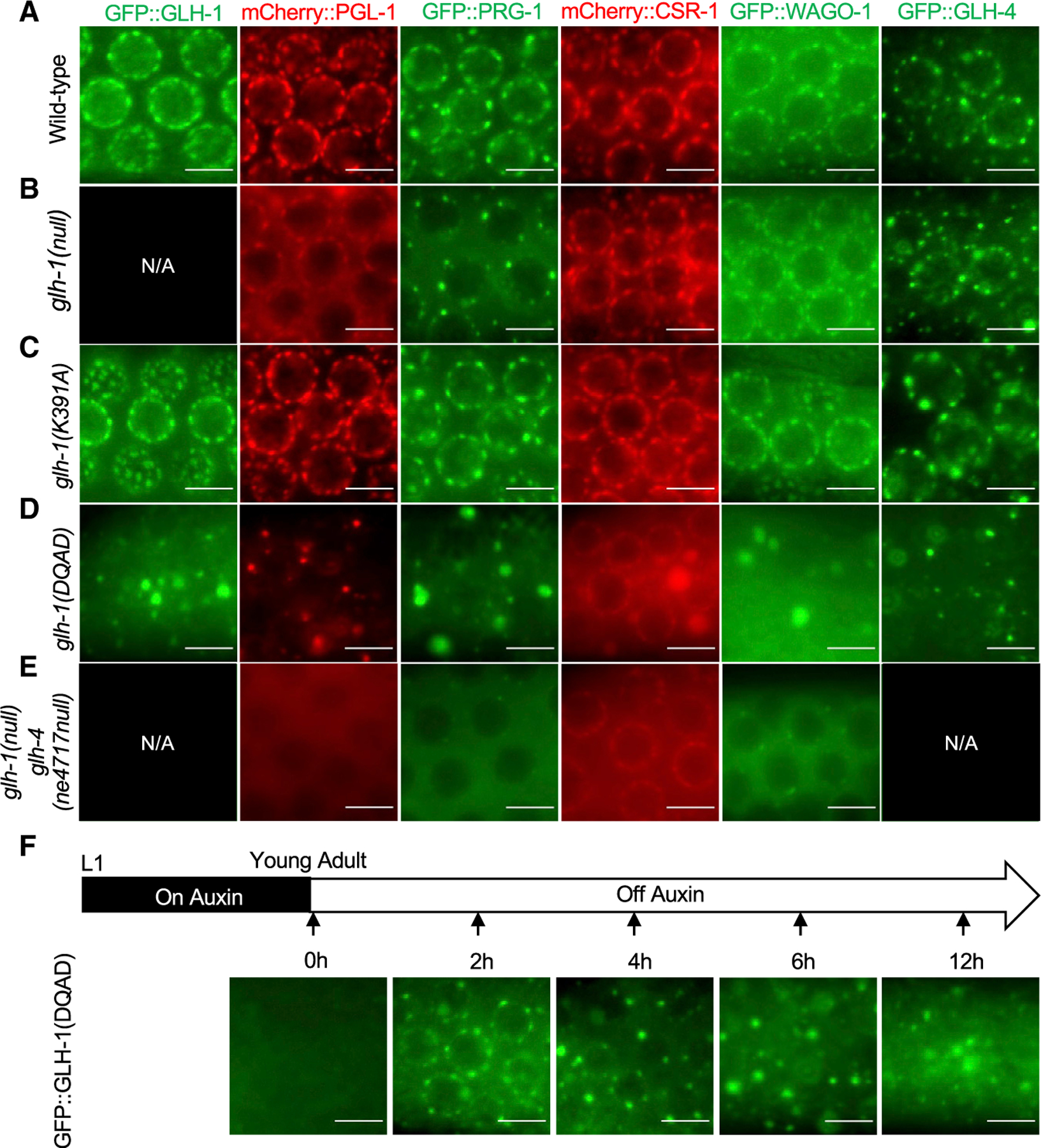
GLH-1 lesions affect the localization of P-granule components (A–E) Fluorescence microscopy showing the subcellular localization of the indicated P-granule proteins in wild-type or *glh* mutant worms. The coding sequences of fluorescence proteins (GFP or mCherry) were inserted in-frame into the endogenous loci using CRISPR-Cas9 gene editing. N/A, not available. Scale bars, 5 μm. (F) Temporal analysis of GFP:GLH-1(DQAD) localization using auxin-induced degron system. Degron:GFP:GLH-1(DQAD) worms were maintained on auxin until the young adult stage, then removed from auxin and imaged at the indicated time points. Scale bars, 5 μm.

To correlate the silencing phenotypes of *glh* mutations with their effects on GLH protein localization and P-granule integrity, we inserted GFP coding sequences into the endogenous *glh-1* and *glh-4* alleles and analyzed their expression. As expected, both GFP:GLH-1 and GFP:GLH-4 localized to perinuclear P granules (Figure 2A). However, whereas GFP:GLH-1 protein was abundantly expressed throughout the germline, including within the mitotic and meiotic zones, GFP:GLH-4 was expressed primarily within the meiotic region of the germline. (Figure S2A).

To examine the consequences of individual *glh-1* or *glh-4* mutations on GLH protein localization and on other P-granule constituents, we used CRISPR to systematically mutate *glh-1* or *glh-4* in six strains, each expressing a fluorescently tagged P-granule component: GFP:GLH-1, GFP:GLH-4, mCherry: PGL-1, GFP:PRG-1, mCherry:CSR-1, or GFP:WAGO-1 (Figure 2). Each CRISPR allele was confirmed by Sanger sequencing, and for each lesion we analyzed the phenotypes of multiple alleles. We found that precise deletion of *glh-1* only mildly affected localization of other P-granule components (Figure 2B): PRG-1 and PGL-1 localized in perinuclear foci (nuage) that resembled wild-type P granules but appeared to be slightly reduced in their levels within nuage and exhibited increased localization in the cytoplasm. The levels of localization of GLH-4, CSR-1, and WAGO-1 within perinuclear nuage remained similar to wild type. Interestingly, GFP:GLH-1(K391A) was localized at apparently wild-type levels within nuage and did not appear to disrupt the perinuclear localization of other P-granule components (Figure 2C). As previously reported (Chen et al., 2020; Marnik et al., 2019), the GLH-1 ATP-hydrolysis mutant, DQAD, dramatically disrupted the localization of P-granule components, causing them to disperse in the cytoplasm and to form aggregates in the gonadal rachis (Figure 2D). Although also prominently localized in aggregates, CSR-1 was the only Argonaute to form obvious perinuclear foci in the DQAD mutant. In the double mutant *glh-4-*null *glh-1-*null, P-granule components formed fewer or, in the case of CSR-1, less pronounced perinuclear foci and were mainly diffuse throughout the cytoplasm but did not form aggregates (Figure 2E).

To understand how GLH-1(DQAD) forms aggregates, we controlled the expression of GFP:GLH-1(DQAD) by introducing an in-frame, auxin-inducible degron (Zhang et al., 2015). We maintained the worms on auxin so that P granules formed properly, then removed worms from auxin and followed the expression of GFP:GLH-1(DQAD) over time. Initially, the mutant protein localized to perinuclear foci, but within 4 h of auxin removal, the number of perinuclear GFP:GLH-1(DQAD) foci declined while cytoplasmic aggregates accumulated (Figure 2F). Thus, although GLH-1(DQAD) initially localizes in perinuclear granules that appear wild-type, continued expression of GLH-1(DQAD) causes the gradual cytoplasmic aggregation of several P-granule components over time.

### *Glh-1* mutants alter secondary small-RNA levels on mRNA targets

**Figure 3.**
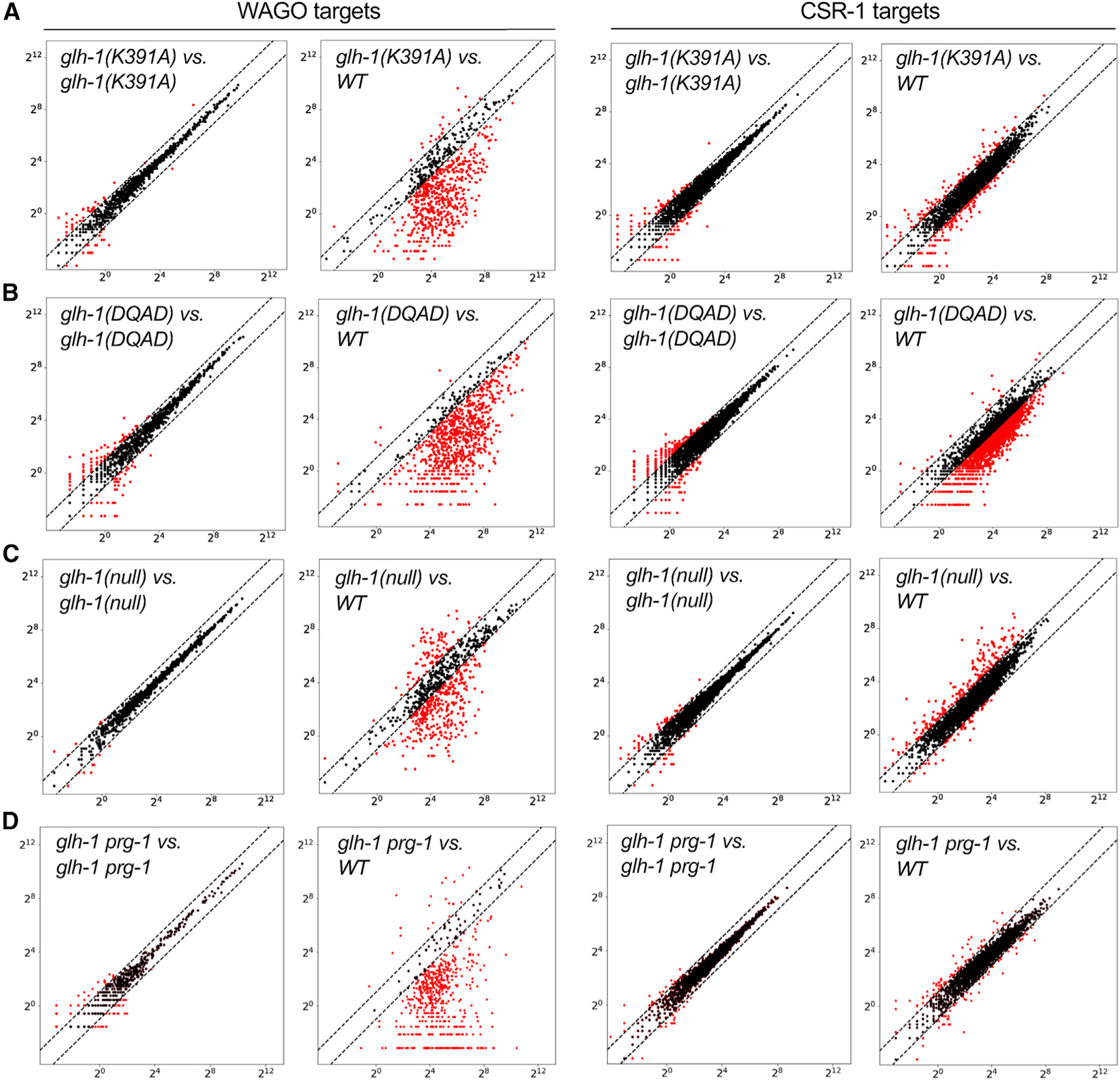
*glh-1* mutants alter secondary small-RNA levels on mRNA targets Scatterplots comparing the levels of small RNAs on WAGO or CSR-1 targets in wild-type (WT) with those in (A) *glh-1*(*K391A*), (B) *glh-1*(*DQAD*), (C) *glh-1-*null, or (D) *glh-1-*null *prg-1* mutants. In (D), the degron tag coding sequence was fused to the *prg-1* locus to deplete PRG-1 from *glh-1-*null worms in the presence of auxin. Each dot represents normalized total 22G-RNA reads per million for a WAGO or CSR-1 target gene. Red dots represent genes whose small RNAs increase or decrease in abundance by at least 2-fold in the mutant compared with the wild type. Dotted lines indicate 2-fold threshold.

To understand how the GLH proteins regulate small-RNA pathways, we used high-throughput sequencing to analyze small-RNA populations in *glh* mutant worms. Although GLH-1 has been reported to interact with DCR-1 (Beshore et al., 2011), which is required for microRNA biogenesis (Grishok et al., 2001; Hutvagner et al., 2001; Ketting et al., 2001), we did not observe defects in microRNA levels (Figure S3A). Consistent with their stronger defects in silencing, fertility, and embryo viability, GLH-1(K391A) and GLH-1(DQAD) mutants showed the strongest effects on small-RNA levels. Many WAGO 22G-RNAs were strongly depleted in the GLH-1(K391A) mutant, whereas piRNAs and CSR-1 22G-RNAs appeared unaffected (Figures 3A and S3C). Expression of GLH-1(DQAD) protein beginning from the L1 stage (i.e., using the degron allele) caused the most severe small-RNA defects: piRNAs as well as WAGO and CSR-1 22G-RNAs were all strongly depleted (Figures 3B and S3C). Expressing GLH-1(DQAD) in young adults caused a mild defect during the first several hours after auxin removal, but by 12 h, piRNA levels were reduced by 70%, WAGO 22G-RNAs by 30%, and CSR-1 22G-RNAs by 10% (Figure S3E). Taken together, these results suggest that GLH-1(K391A) disrupts silencing on WAGO targets, whereas the GLH-1(DQAD) mutant disrupts all the Argonaute small-RNA pathways that localize to P granules, perhaps by causing P-granule aggregation (see discussion).

*glh-1-*null mutants did not alter piRNA levels (Figure S3C) but caused complex changes in small-RNA levels for germline genes, including WAGO and CSR-1 targets (Figures 3C and S3D). The levels of 22G-RNAs targeting previously annotated CSR-1 targets were increased relative to wild type in many cases, while 22G-RNA levels were increased on some previously annotated WAGO targets and decreased on others (Figure 3C); these changes were reproducible in the biological replicates (Figures 3C and S3B, and see below). Although *glh-1 prg-1* double nulls are sterile, we were able to conditionally remove PRG-1 from *glh-1-*null animals using an auxin-inducible *degron*::*prg-1* allele constructed at the endogenous *prg-1* locus. Exposing these animals to auxin for 24 h from the L4 stage greatly reduced the number of genes with increased 22G-RNA levels for both the WAGO and CSR-1 targets (Figure 3D).

**Figure 4.**
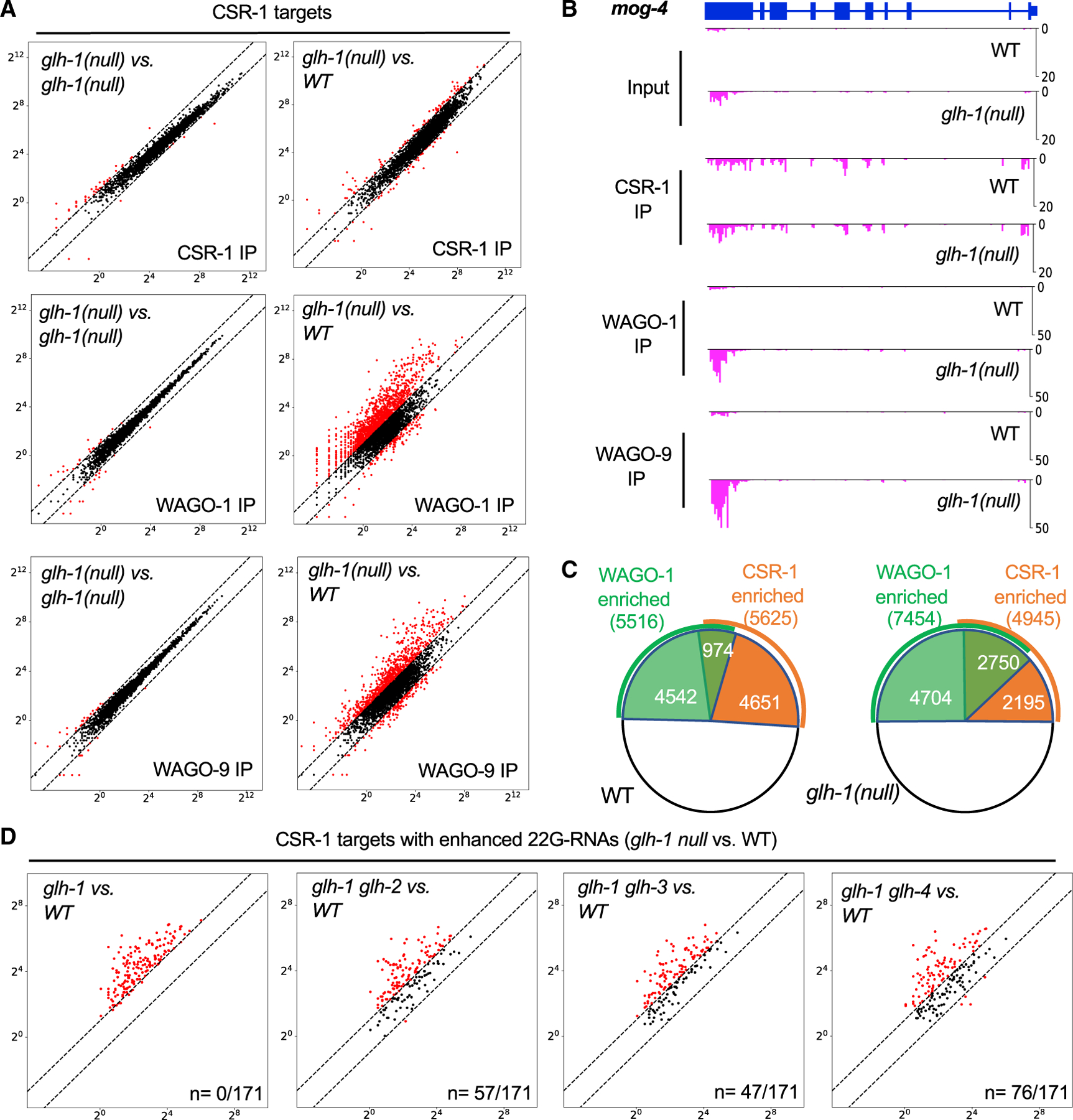
GLH paralogs promote ectopic WAGO 22G-RNA biogenesis (A) Scatterplots comparing the levels of small RNAs on CSR-1 targets recovered in CSR-1, WAGO-1, and WAGO-9 IPs cloned from wild-type (WT) or *glh-1-*null worms, as in Figure 3. (B) Genome browser view of *mog-4* 22G-RNAs in CSR-1, WAGO-1, and WAGO-9 IPs from WT and *glh-1-*null animals. y axis, normalized number of 22G-RNAs reads per million. (C) Pie charts depicting the numbers of genes whose small RNAs are enriched by CSR-1 or WAGO-1 IPs from WT or *glh-1-*null worms. (D) Scatterplots showing suppression of ectopic 22G-RNAs on 171 CSR-1 targets in *glh-1*-null mutant by depleting GLH-2, GLH-3, or GLH-4. The degron tag coding sequence was fused to the *glh-2*, *glh-3*, or *glh-4* locus to deplete each GLH paralog from *glh-1-*null worms in the presence of auxin.

To determine which Argonautes associate with the ectopic 22G-RNAs produced *in glh-1-*null animals, we sequenced small RNAs that co-immunoprecipitated with WAGO-1, WAGO-9, or CSR-1 (Figure 4A). These IP studies confirmed that some WAGO targets made higher levels of WAGO-1- and WAGO-9-bound 22G-RNAs and others made fewer. Strikingly, however, these data revealed that many CSR-1 targets exhibit markedly increased levels of 22G-RNAs that are bound to WAGO Argonautes (Figure 4A). For example, wild-type and *glh-1-*null worms produce the same level of CSR-1-bound *mog-4* 22G-RNAs, but *glh-1-*null worms exhibited a dramatic increase in *mog-4* 22G-RNAs bound to WAGO-1 and WAGO-9 (Figure 4B).

To gain a more comprehensive picture of how *glh-1-*null animals affect Argonaute targeting, we defined sets of genes enriched for binding to the CSR-1 and WAGO-1 Argonautes in our wild-type and mutant IP datasets. We calculated the number of reads per million (RPM) mapping to each gene in the input and IP datasets. A gene was scored as enriched if the RPM level in the IP increased by 2-fold over the level in the corresponding input dataset. In wild-type animals, approximately equal numbers of germline genes were enriched in the WAGO-1 and CSR-1 IPs (5,516 and 5,625, respectively, Figure 4C). Approximately 1,000 genes were enriched in both IPs from wild-type worms. Strikingly, in *glh-1-*null animals the number of WAGO-1-enriched genes increased by 35% to 7,454, while the number of CSR-1-enriched genes declined slightly. The percentage of genes enriched in both CSR-1 and WAGO-1 IPs increased from 17% in wild type to 56% in the *glh-1* mutant (Figure 4C). A similar change in targeting was observed for WAGO-9/HRDE-1-associated 22G-RNAs. Thus, in *glh-1*-null animals WAGO targeting increases on thousands of germline mRNAs.

### GLH paralogs promote ectopic WAGO 22G-RNA biogenesis

In the absence of GLH-1, other GLH paralogs might promote the production of aberrant 22G-RNAs on target mRNAs. To test this idea, we identified 171 CSR-1 targets showing increased 22G-RNA levels (Table S2). We then confirmed that the ectopic small RNAs produced on these genes were enriched in WAGO-1 IP or WAGO-9 IP but not in CSR-1 IP (Figure S4A). To ask whether the ectopic small RNAs depend on GLH-2, GLH-3, or GLH-4, we inserted degron tags into each of these genes in a *glh-1*-null mutant strain and then monitored small-RNA levels by sequencing after several hours of auxin exposure. In two biological replicates for each strain, we found that auxin-mediated depletion of GLH paralogs suppressed ectopic 22G-RNAs from more than half of the mistargeted genes (106 out of 171; Figures 4D and S4C). For example, exposing *degron*::*glh-4 glh-1-*null double mutants to auxin dramatically reduced ectopic 22G-RNAs at 76 of the 171 mistargeted genes (Figure 4D). Unlike *glh-1-*null mutants, *glh-1*(*K391A*) did not exhibit ectopic 22G-RNAs on CSR-1 targets (Figure S4B). Taken together, these findings suggest that, in the absence of GLH-1 protein, its paralogs promote the mistargeting or overamplification of WAGO 22G-RNAs. GLH-1(K391A) prevents WAGO 22G-RNA induction on piRNA-dependent WAGO targets but also prevents mistargeting, perhaps by competing with the GLH paralogs for downstream WAGO 22G-RNA amplification and silencing machinery (see below).

Deleting two or more *glh* paralogs causes sterility, but we were able to use small-RNA sequencing data from the pairwise degron doubles, described above, to create a composite list of genes with GLH-paralog-dependent 22G-RNAs. Collectively, summing the genes depleted at least 2-fold in 22G-RNAs in each double mutant versus wild type identified 2,000 genes. The majority of these genes (70%) were among the 1,825 genes depleted 2-fold or more of WAGO 22G-RNAs in *glh-1*(*K391A*), further supporting the idea that GLH-1(K391A) blocks the ability of GLH paralogs to promote WAGO 22G-RNA biogenesis (Figure S4F).

### GLH-1(K391A) exhibits enhanced binding to WAGO-1

**Figure 5.**
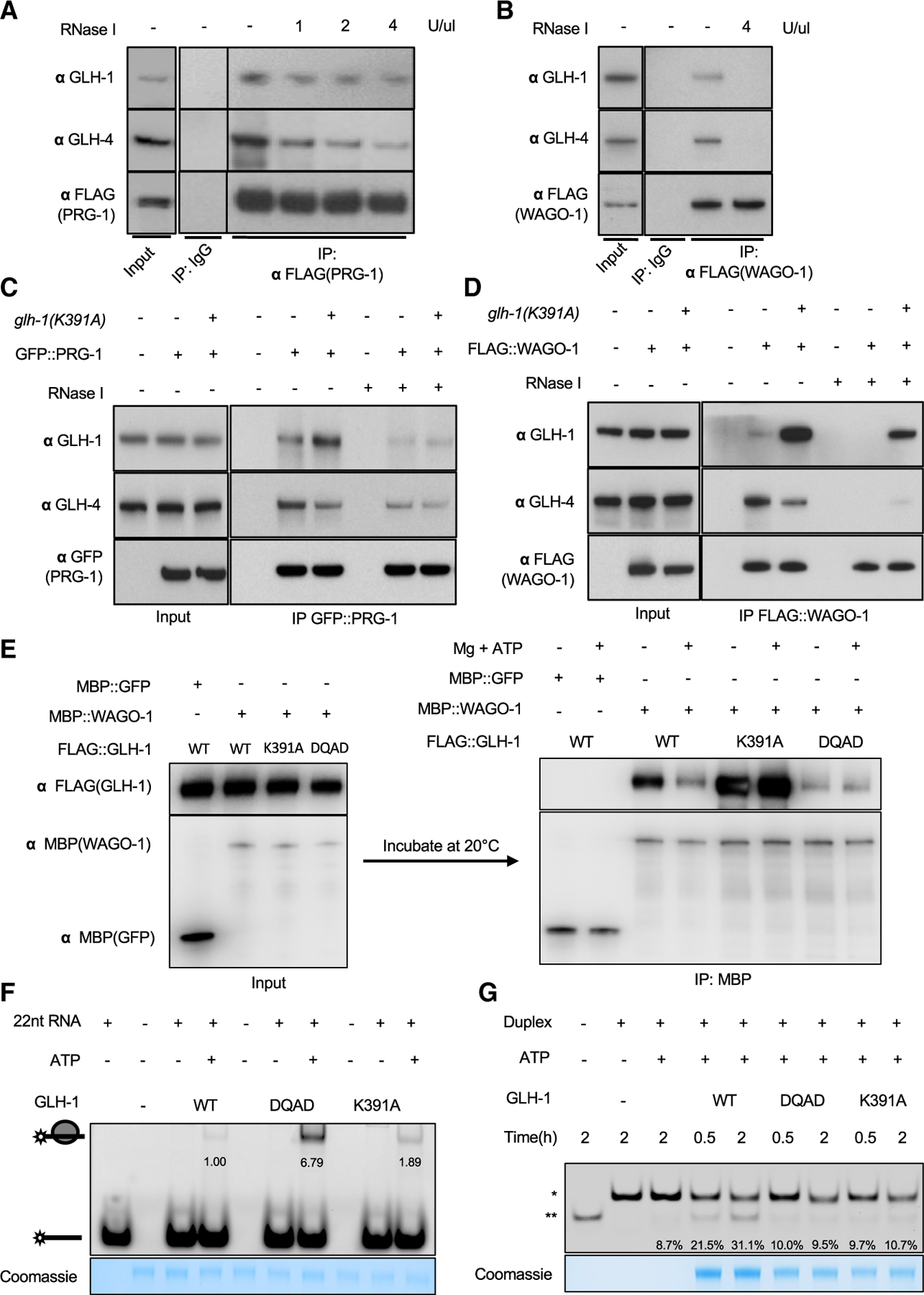
GLH-1(K391A) exhibits enhanced binding to Argonautes both *in vivo* and *in vitro* (A and B) Western blot analyses of co-IP experiments showing that PRG-1 (A) and WAGO-1 (B) physically interact with GLH-1 and GLH-4. Lysates from *FLAG*::*prg-1* or *FLAG*::*wago-1* worms were incubated with mouse IgG or FLAG-M2 antibodies in the absence (−) or presence of RNase I (concentration indicated). Blots were probed with GLH-1, GLH-4, or FLAG-M2 antibodies. (C) Western blot analysis of co-IP experiment showing PRG-1 interactions with GLH-1 or GLH-4 in wild-type (−) or *glh-1*(*K391A*) mutant (+) worms. Samples treated with RNase I indicated by +. GFP:PRG-1 was immunoprecipitated using GFP nanobody, and eluted fractions were resolved by SDS-PAGE. Blots were probed with anti-GLH-1, GLH-4, or GFP antibodies. (D) Western blot analysis of co-IP experiment showing WAGO-1 interactions with GLH-1 or GLH-4 in wild-type (−) or *glh-1*(*K391A*) mutant (+) worms, as in (C). (E) *In vitro* interactions between MBP:WAGO-1 and FLAG:GLH-1. MBP:WAGO-1 (or MBP:GFP) and FLAG:GLH-1 (WT or mutants) were expressed in *E*. *coli*. Lysates containing MBP:WAGO-1 (or MBP:GFP) were combined with the lysates containing FLAG:GLH-1 (WT or mutants). MBP fusions were captured on MBP affinity resin and incubated with Mg^2+^ and ATP at 20°C for 20 min. Complexes were resolved by SDS-PAGE and analyzed by western blot using anti-FLAG or anti-MBP antibodies. Presence (+) or absence (−) of Mg and ATP are indicated. (F) Gel-shift analyses showing binding of synthetic RNA oligos by recombinant GLH-1 (WT, DQAD, or K391A) proteins. Recombinant GLH-1 was incubated with 22-nt 5′ FAM-labeled ssRNA for 30 min at room temperature. Samples were resolved by native PAGE and visualized using a Bio-Rad imager. (G) *In vitro* unwinding assays. Recombinant GLH-1 (WT, DQAD, or K391A) proteins were incubated with 11-nt-long RNA duplex with 11-nt 3′ extension for 0.5 or 2 h. Products were resolved by native PAGE, and fluorescence signals were visualized using a Bio-Rad imager. Single asterisk indicates RNA duplex, and double asterisk unwound ssRNAs.

IP-western blot assays confirmed that GLH-1 and GLH-4 co-precipitate with PRG-1 and WAGO-1 (Figures 5A and 5B). Pretreating the lysate with ribonuclease I (RNase I) reduced but did not eliminate the recovery of GLH proteins in the PRG-1 IP (Figures 5A and S5B), suggesting that interactions with PRG-1 are only partially bridged by RNA. By contrast, RNase pretreatment greatly reduced the interactions between the GLH proteins and WAGO-1 (Figures 5B and S5C), suggesting that these interactions depend more strongly on RNA bridging.

To ask whether the stronger-than-null phenotypes of GLH-1(K391A) reflect changes in its protein interactions, we used mass spectrometry to identify proteins that co-precipitate with wild-type GLH-1 or with GLH-1(K391A). This analysis revealed that the K391A lesion does not significantly perturb GLH-1 interactions with other GLH proteins. We were intrigued to note, however, that PRG-1 and WAGO-1 were enriched in GLH-1(K391A) immunoprecipitates (Figure S5A). We confirmed these results using co-IP studies (Figures 5C, 5D, S5D, and S5E). The amount of WAGO-1 associated with GLH-1 protein increased dramatically in K391A mutants when compared with wild type, and a portion of this interaction resisted RNase treatment (Figures 5D and S5E). Whereas in wild type both PRG-1 and WAGO-1 interacted more strongly with GLH-4 than with GLH-1, in K391A mutants they preferred GLH-1(K391A) over GLH-4 (Figures 5C and 5D). We also noted that in GLH-1(K391A) lysates, when compared with wild type, the interaction between WAGO-1 and GLH-4 appeared to become partially resistant to RNase treatment (Figures 5D and S5E).

The above findings suggest that ATP binding by GLH-1 regulates a direct (RNA-independent) interaction with WAGO-1. To explore this possibility, we tagged WAGO-1 with the maltose-binding protein (MBP) and performed *in vitro* binding assays between WAGO-1 and GLH-1 proteins. We used MBP:GFP as a negative control for specificity. Lysates containing MBP:WAGO-1 or MBP:GFP were mixed with lysates containing wild-type or mutant GLH-1 protein and incubated with or without ATP at 20°C for 20 min. In MBP pull-downs, MBP:WAGO-1 co-precipitated more GLH-1(K391A) than wild-type GLH-1 (Figure 5E), consistent with IPs from worm lysates. Adding magnesium and ATP prior to MBP pull-down slightly reduced WAGO-1 binding to wild-type GLH-1 but did not affect binding to GLH-1(K391A) or GLH-1(DQAD) (Figure 5E). The control MBP:GFP fusion protein did not bind to GLH-1 (Figure 5E). These data suggest that WAGO-1 preferentially interacts with GLH-1 when the helicase domain is in an ATP-unbound conformation.

### GLH-1 has RNA binding and unwinding activities *in vitro*

We next asked whether GLH-1 can bind RNA and unwind RNA duplexes *in vitro*. To check for RNA binding, we incubated wild-type or mutant GLH-1 proteins with fluorescently labeled 22-nt single-stranded RNA (ssRNA) oligo and visualized resulting protein/RNA complexes as gel shifts by native PAGE. We found that both GLH-1 wild type and K391A showed weak binding to the ssRNA oligo, whereas GLH-1(DQAD) showed strong ATP-dependent binding (Figure 5F).

To examine unwinding activity, we incubated recombinant GLH-1 proteins with Mg^2+^ and ATP and a short fluorescently labeled duplex RNA with an 11-nt 3′ single-strand extension. In the presence of excess single-stranded competitor lacking the 11-nt extension, unwound labeled molecules will anneal to the competitor to produce a labeled product that can be distinguished from the substrate by PAGE. Indeed, the shorter labeled product accumulated over time in the presence of wild-type GLH-1 protein (Figure 5G). As expected, unwinding required ATP and was reduced by the K391A and DQAD mutations (Figure 5G).

### GLH-1 and GLH-4 associate with overlapping sets of WAGO target mRNAs

To determine whether GLH proteins bind RNA *in vivo*, we used a modified CLIP assay (Chi et al., 2009; Nostrand et al., 2016) to analyze four proteins—GLH-1(wild-type [WT]), GLH-1(K391A), DEGRON:GLH-1(DQAD), and GLH-4(WT)—all expressed as FLAG epitope fusions from the endogenous *glh* loci. To measure background binding to the matrix, adult worms expressing FLAG-tagged GLH proteins were analyzed in parallel with control strains expressing the corresponding untagged GLH proteins. Populations of DEGRON:GLH-1(DQAD) worms were grown to adulthood in the presence of auxin to deplete the toxic protein, after which auxin was removed to allow GLH-1(DQAD) to accumulate in P granules for either 2 h or 4 h before crosslinking.

**Figure 6.**
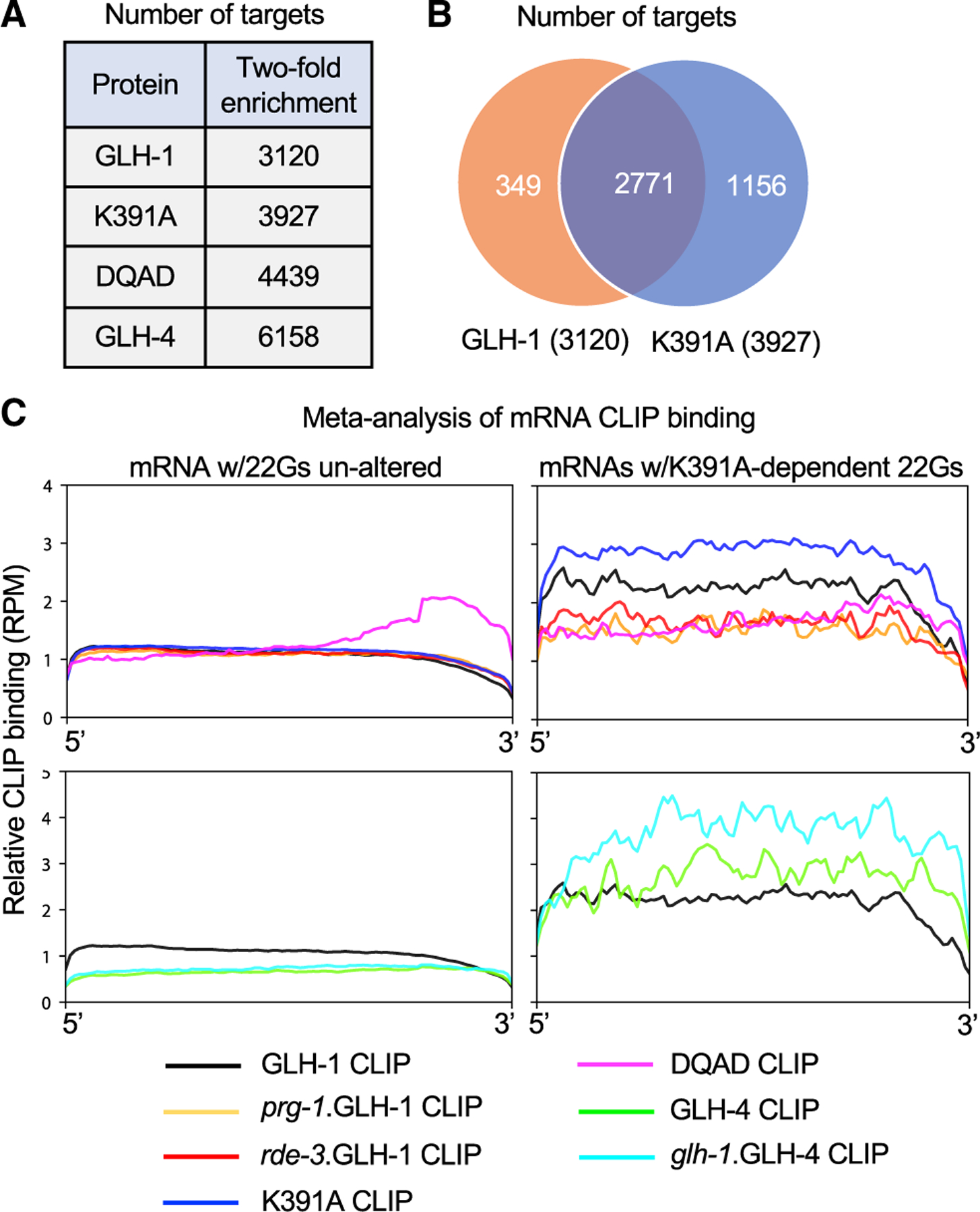
GLH-1 and GLH-4 associate with overlapping sets of WAGO target mRNAs (A) Table depicting the number of genes whose mRNAs are enriched at least 2-fold in GLH-1, GLH-1(K391A), GLH-1(DQAD), and GLH-4 CLIP compared with controls. (B) Venn diagram showing the overlap between GLH-1 (orange) and GLH-1 K391A (blue) enriched CLIP targets. (C) Metagene analysis of GLH-1 or GLH-4 binding on mRNAs whose 22G-RNAs were depleted in *glh-1*(*K391A*) worms (2-fold reduction and >5 RPM) compared with control mRNAs whose 22G-RNA levels were unaffected in *glh-1*(*K391A*) worms. Samples included: GLH-1 CLIP in WT (black), *prg-1* (yellow), or *rde-3* (red) worms; GLH-1(K391A) CLIP (blue), GLH-1(DQAD) CLIP (magenta); and GLH-4 CLIP in WT (green) or *glh-1-*null (turquoise) worms. y axis indicates averaged CLIP reads along each mRNA as a percentage of the mRNA length. The aggregate binding to each interval was plotted from 0% (5′) to 100% (3′) along the length of the transcript.

Using a 2-fold enrichment over background binding, CLIP-sequencing analyses identified 3,120 GLH-1(WT), 3,927 GLH-1(K391A), 4,439 GLH-1(DQAD), and 6,158 GLH-4 targets (Figure 6A). The majority of RNA fragments associated with these proteins derived from mRNAs (Figure S6A). piRNAs were also recovered at high levels, especially in GLH-4 IP complexes where they made up 25% of the reads; by comparison, piRNAs constituted 5%, 3%, and 1% of reads in GLH-1(WT), GLH-1(K391A), and GLH-1(DQAD) complexes (Figure S6A). Although we expected K391A to disrupt RNA binding, we were surprised to find that K391A CLIP enriched nearly 30% more mRNAs than wild type (Figures 6A and 6B). Moreover, GLH-1(K391A) enriched 89% of the targets enriched by GLH-1(WT) (Figure 6B).

To explore the relationship between GLH–mRNA binding and 22G-RNA levels, we analyzed the subset of mRNAs whose 22G-RNA levels decreased by at least 2-fold in GLH-1(K391A) mutants. We chose this group of targets because the K391A mutant strongly depletes a subset of PRG-1-induced WAGO 22G-RNAs that depend on GLH-1 and/or its paralogs (i.e., GLH-dependent targets) without eliminating scaffolding functions required for other P-granule-associated Argonaute pathways. We used the remainder of germline mRNAs as a control set. To reveal how CLIP binding distributes along the length of mRNAs, we performed a meta-analysis of GLH binding along GLH-dependent or control mRNAs (see STAR Methods). As expected, wild-type GLH-1 was enriched on GLH-dependent targets but not on the control set (Figure 6C, top panel). Moreover, enriched binding required PRG-1 and RDE-3 activities, consistent with the idea that direct binding of wild-type GLH-1 correlates with GLH-dependent 22G-RNA production on these targets. Compared with wild-type GLH-1, GLH-1(K391A) showed enhanced binding along these same targets (Figure 6C, top panel).

GLH-1(DQAD), in contrast, showed a strikingly different pattern of mRNA binding. At 2 h after auxin removal, when GLH-1(DQAD) was localized in perinuclear nuage (Figure 2F), DQAD primarily enriched mRNAs (79% of CLIP reads). Notably, however, the mRNAs enriched by DQAD were not restricted to GLH-dependent targets but also included many GLH-1-independent targets, including CSR-1 targets. Moreover, DQAD preferred to bind the 3′ regions of these mRNAs (Figure 6C, top panel).

Consistent with competition between GLH-1 and GLH-4 for WAGO 22G-RNA induction, GLH-4 binding to GLH-1-dependent target mRNAs was increased in a *glh-1-null* mutant (Figure 6C, bottom panel). In a separate meta-analysis, we found that sites of ectopic 22G-RNAs in *glh-1* mutants correlate with ectopic or increased GLH-4 binding (Figure S6B). Taken together, these findings suggest that RNA binding by GLH-1 and GLH-4 correlates with the production of PRG-1- and RDE-3-dependent WAGO-pathway small RNAs.

## DISCUSSION

### A link between the GLH-1 ATP cycle and Argonaute surveillance

A poorly understood aspect of transgenerational silencing is how targeting by a primary Argonaute (PRG-1 for piRNAs and RDE-1 for the dsRNA response) leads to the amplification of heritable secondary small RNAs. Here, we have shown that GLH proteins—members of an expanded family of DEAD-box proteins related to *Drosophila* VASA—physically interact with Argonaute proteins and with target RNA to promote transgenerational silencing. RNA binding by the helicase domain of DEAD-box proteins is gated by ATP binding, while release requires ATP hydrolysis (Yang and Jankowsky, 2005; Liu et al., 2008; Xiol et al., 2014). Surprisingly, a lesion expected to prevent ATP binding, GLH-1(K391A), failed to prevent *in vitro* RNA binding and instead caused enhanced association with Argonaute pathway target mRNAs *in vivo*. A glutamate to aspartic acid lesion in the DEAD-box (motif II), GLH-1(DQAD), exhibited enhanced RNA binding *in vitro* (as expected) and exhibited Argonaute-non-specific association with the 3′ ends of mRNAs *in vivo*.

Our findings are consistent with previous work implicating a role for GLH proteins in the transmission of silencing signals to offspring (Spracklin et al., 2017). GLH-1(K391A) was strongly defective in inheritance of silencing but fully sensitive to RNAi in exposed animals. Although *glh-1*-null mutants showed a csRNAi defect in the exposed generation, which correlated with overproduction of small RNAs on endogenous genes, this defect was suppressed by removing *prg-1* activity. Thus, our findings support a model in which GLH proteins are not general factors required for amplification of the silencing signal but rather function more directly to promote transgenerational silencing. Perhaps upstream Argonautes, including RDE-1, PRG-1, and transgenerationally inherited WAGOs, recruit GLH proteins to bind their mRNA targets within nuage to initiate transgenerational silencing. Initial binding of GLH proteins could serve to retain target mRNA in nuage or to promote its conversion to template RNA required for amplification of transgenerational signals that are transmitted along with nuage to offspring.

**Figure 7.**
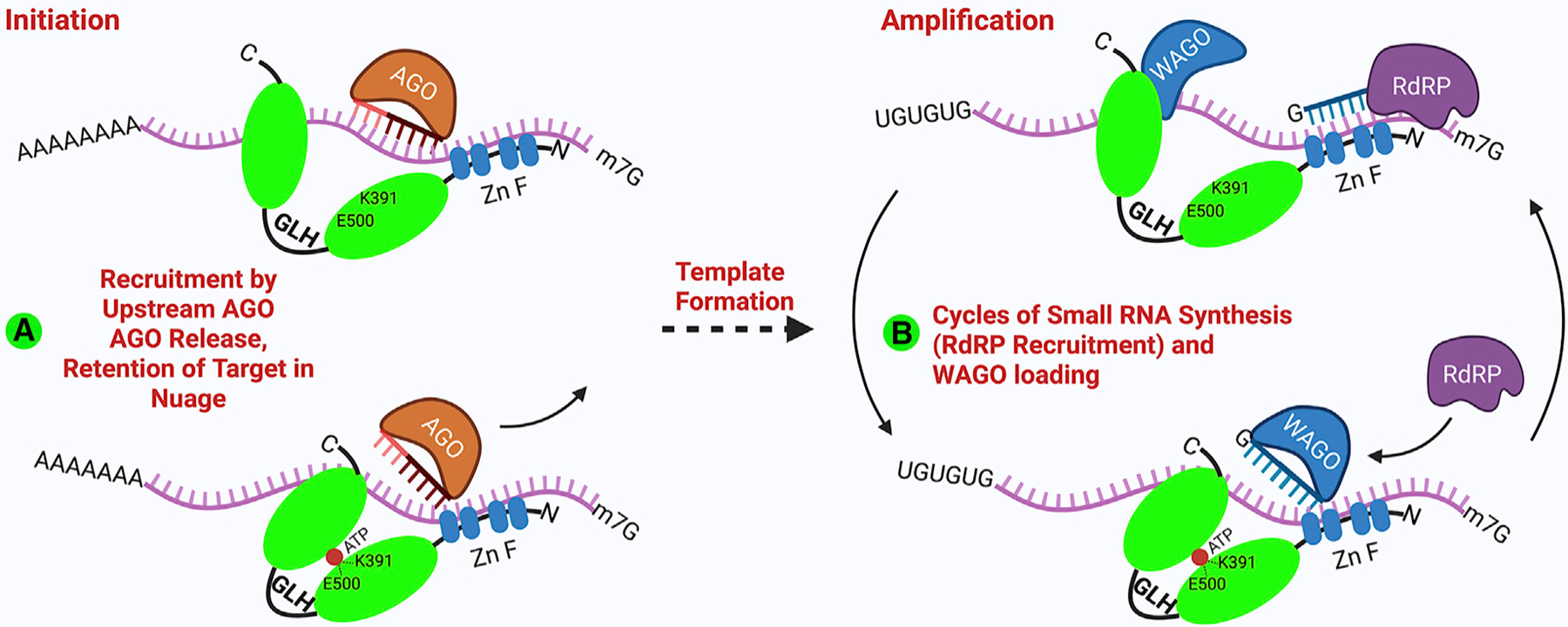
Model illustrating possible roles for GLH-1 in promoting transgenerational silencing GLH-1 is recruited to target mRNA within nuage by an upstream Argonaute; once bound the GLH protein could use its helicase activity to help the upstream Argonaute release to find new targets (A). GLH-1 could then remain bound to template RNA, retaining it within nuage to promote successive rounds of *de novo* small-RNA synthesis and WAGO loading (B). Together, these activities could program the nuage-localized and nuclear-localized WAGO Argonautes that promote transgenerational inheritance.

Like other DEAD-box proteins (Yang and Jankowsky, 2005), GLH-1 can bind and remodel short RNA duplexes upon ATP hydrolysis. Nevertheless, GLH-1 also binds RNA independently of ATP binding and presumably, therefore, independently of its helicase domain, which might allow GLH-1 complexes to remain bound through multiple cycles of target remodeling. Moreover, in its ATP-unbound form GLH-1 preferentially associates with WAGO-1, a downstream Argonaute that localizes in nuage. Thus, perhaps GLH binding retains the target RNA in nuage while positioning the helicase to promote the release of the upstream Argonaute (Figure 7, model A) or to mark the template RNA for multiple cycles of RdRP recruitment and downstream WAGO loading (Figure 7, model B).

Nuage is a complex and crowded molecular environment where numerous protein-protein and protein-RNA interactions occur within a number of nuage subdomains. Localization studies suggest that Argonautes (and hence the initial surveillance step) reside in the largest subdomain of nuage (the P-granule domain), while many components required for amplification of silencing signals reside in smaller subdomains known as Mutator foci (Philips et al., 2012; Zhang et al., 2011). Our findings suggest that GLH-1 couples RNA surveillance to small-RNA amplification required for transgenerational inheritance and would therefore predict that GLH-1 resides (at least transiently) in both the P-granule and Mutator foci. GLH-1 has long been known to localize in P granules (Roussell and Bennett 1993; Gruidl et al., 1996), but whether or not the protein also co-localizes in Mutator foci has not been addressed. It is intriguing that paralogs of a distinct family of DEAD-box proteins, including MUT-14 and SMUT-1, co-localize in Mutator foci and like their GLH homologs are known to exhibit dynamic and partially redundant functions required for small-RNA amplification (Philips et al., 2014). Whether or not GLH proteins remain engaged with target mRNA during amplification within Mutator foci, or perhaps hand the target off to other co-factors in Mutator foci, will require further localization and biochemical studies.

In insects and mammals, Piwi Argonautes amplify epigenetic signals on transposon RNAs via an amplification cycle in which the cut site of a loaded Argonaute creates the 5′ end of a guide RNA that is loaded onto a downstream Argonaute. This downstream Argonaute can then cleave a corresponding anti-sense transcript to regenerate the upstream guide, and so on. Interestingly, this ‘‘ping-pong’’ amplification cycle requires the DEAD-box protein VASA, and studies in insect cells have linked VASA ATP binding with formation of a Piwi complex, called the ‘‘amplifier complex’’ (Xiol et al., 2014). A VASA-K230 N mutation analogous to GLH-1(K391A) disrupts binding to the PIWI orthologs (Siwi and AGO-3) that mediate the ping-pong cycle. In contrast, the VASA(DQAD) mutant interacted with both Piwi Argonautes, albeit bridged by RNA. Interestingly, this VASA(DQAD) complex contained Siwi-associated piRNAs but not AGO3-associated piRNAs, suggesting that the mutant VASA(DQAD) protein traps an intermediary complex in the ping-pong cycle, prior to loading the downstream AGO3 Argonaute. Although the details differ, these findings and those from the present study hint at a conserved role for the ATPase activities of VASA and GLH-1 in the amplification of small-RNA silencing.

### Conclusion

Here we have shown that the VASA homologs, the GLH proteins, which are required for P-granule assembly and homeostasis (this study; Chen et al., 2020; Spike et al., 2008a), play complex and dynamic roles in Argonaute surveillance. Although the GLH paralogs have often been considered redundant factors, our studies reveal major differences in their mRNA, small-RNA, and protein interactions. For example, our analysis suggests that GLH-1 competes with or prevents recruitment of GLH paralogs on many target mRNAs. Thus, mutating one GLH factor causes mistargeting of the other. Mistargeting of GLH paralogs in turn requires PRG-1 and results in expression of ectopic WAGO pathway small RNAs on thousands of germline mRNAs, including many CSR-1 pathway targets. Mistargeting of the WAGO pathway to thousands of additional mRNAs likely explains the cold-sensitive defect of *glh-1*-null mutants, as ectopic piRNA-dependent WAGO silencing could reduce availability of WAGO machinery to function in canonical RNAi induced by exogenous dsRNA. Alternatively, piRNA-dependent mistargeting could silence one or more genes that encode protein effectors of WAGO silencing, as suggested in recent studies by Ouyang et al. (2019) and Dodson and Kennedy (2019). Our findings reveal GLH proteins as RNA-binding factors that impose delicate regulation on mRNA homeostasis and expression within nuage. Understanding the cascading effects of mutations that shift the balance of RNA binding and surveillance in nuage could shed light on related perturbations in RNA-binding factors that cause myriad human disorders.

### Limitations of the study

In this study we used a combination of genetics, microscopy, and biochemical methods to explore the functions of GLH family members. The GLH proteins are essential, and null alleles cause synthetic phenotypes with pleiotropic effects on multiple germline Argonaute pathways. Redundancy and synthetic sterility make it difficult to assign specific genetic functions to the GLH family members. The absence of phenotypes when the zinc-finger domain is deleted from GLH-1, for example, could be masked by redundant activities provided by the zinc-finger domains of GLH paralogs. On the other hand, strong phenotypes might result indirectly from misregulation of unknown factors. The special alleles of GLH-1 create proteins that could adopt novel properties or interfere with pathways that are unrelated to the normal wild-type activities of the protein. Our microscopy studies reveal patterns of localization that in some cases appear similar to, and in others are dramatically distinct from wild-type localizations. However, we cannot conclude that the observed phenotypic and molecular changes are caused by the altered localizations; for example, the absence of nuage or its aggregation might cause defects in small-RNA levels, or vice versa, or they could be merely correlated and not causal. Similarly, just because the localization of GLH-1(K391A) appears similar to its wild-type localization, we cannot rule out changes in other nuage factors that associate with it; for example, GLH-1(K391A) might reside in a functionally distinct domain of nuage that looks similar to wild-type P granules. Additionally, the biochemical studies reveal interactions that occur within a subset of GLH complexes that are soluble under the extraction conditions used to prepare lysate. Thus, the IP-MS studies and western blotting assays are likely to miss significant interactions that occur in complexes excluded from the analyses. Finally, assays on bacterially purified proteins will unavoidably lack endogenous co-factors or covalent modifications that regulate RNA binding and unwinding properties of the proteins analyzed.

## STAR★METHODS

### RESOURCE AVAILABILITY

#### Lead contact

Further information and requests for resources and materials should be directed to and will be fulfilled by the lead contact, Craig Mello (Craig.Mello@umassmed.edu).

#### Materials availability

All materials generated in this study are available from the lead contact without restrictions.

#### Data and code availability

- Original and processed small-RNA and CLIP deep sequencing datasets are publically available under the accession number GEO: GSE195536 and GSE198101.
- This study did not generate any new code, but the scripts used in the study are available from the lead contact upon request.
- Any additional information required to reanalyze the data reported in this paper is available from the lead contact upon request.

### EXPERIMENTAL MODEL AND SUBJECT DETAILS

*C*. *elegans* strains were grown on NGM agar plates seeded with *E*. *coli* OP50, essentially as describe (Brenner, 1974) for regular use, or with *E*. *coli* HT115 for RNAi assays (Kamath and Ahringer, 2003). Strains used in this study were generated either by cross or via CRISPR-cas9 method. The information of worm strains are listed in Table S3.

### METHOD DETAILS

#### Immunoprecipitation and mass spectrometry

100,000 synchronized *flag*::*tev*::*prg-1 and flag*::*tev*::*wago-1* worms were homogenized by FastPrep-24 (MP Biomedicals) in lysis buffer (20 mM HEPES PH = 7.0, 250 mM Sodium Citrate, 0.5% Triton X-100, 0.1% Tween 20, 1 mM DTT). The same experimental procedures were also performed for N2 worms as negative controls. Protein extracts were incubated with 3ul anti-FLAG antibody (Clone M2, Sigma-Aldrich) and 50ul Protein-G magnetic beads (Thermo Scientific) at 4°C for 2 h. Prior to elution, pull-downed components were pretreated with RNase for removing proteins bridged by RNAs. Immunoprecipitates were released from beads by TEV protease cleavage (Thermo Scientific). Finally, the purified protein complex were resolved by SDS-PAGE and visualized by Silver staining (Pierce). The elutes were precipitated by acetone and air dried. Protein samples were re-suspended and digested with Trypsin. LC-MS/MS were conducted at Mengqiu Dong’s lab in NIBS. MS data were processed as described previously (Feng et al., 2017). Proteins with at-least two fold enrichment of relative peptide levels from IP samples over background (IP from N2) were identified as co-factor of PRG-1 and WAGO-1. See Table S1 for lists of proteins identified in this assay and their descriptions.

#### RNAi Screen

RNAi assays were performed by growing worms on HT115 *E*. *coli* transformed with plasmids expressing either control dsRNA (L4440) or dsRNA targeting 16 tested genes for over 10 generations (Kamath and Ahringer, 2003). Synchronized worms were grown on IPTG-NGM plates at 20°C from L1 stages. More than 60 animals were scored for expression of piRNA sensor (*cdk-1*::*gfp IV*;*21ux anti-gfp X*) in each generation. RNAi that causes lethality was indicated in Figure 1 with asterisk.

#### Gene editing by CRISPR

*C*. *elegans* strains were produced using CRISPR-cas9 RNP methods previously described (Dokshin et al., 2018) by co-injection of Cas-9 RNPs, donors (oligos and melted PCR products) and rol-6 marker. At-least two independent alleles were generated for each strain. Sequences of guide RNAs and donors are listed in Table S4.

#### Desilencing of transgene reporters in *C*. *elegans*

Multiple mutations for *glh-1 and glh-4* genes were introduced to *piRNA sensor*, *cdk-1*::*gfp*;*21ux anti-gfp LGIV* and wago pathway sensor *gfp*::*cdk-1 LGIV* worms by crossing and by CRSIPR-cas9 method. All worms were grown at 20°C and at least 60 worms were scored by Zeiss M2 fluorescence microscopes for each generation. During passaging, over 5 healthy worms were randomly picked to new plates to ensure unbiased results.

#### Assays for assessment of RNAi inheritance and cold-sensitive RNAi

For inheritable RNAi assay, synchronous population of worms were exposed to *gfp* RNAi or L4440 RNAi for one generation, then the adults were bleached and their L1 progenies were plated on regular OP50 food. Fluorescent signals (GFP) in 100 worms were checked for each generation. For cold-sensitive RNAi assay, synchronized animals were fed with *pos-1* RNAi or L4440 RNAi and grown at 15°C, 20°C and 25°C respectively until they laid sufficient eggs for scoring.

#### Fertility of *C*. *elegans*

Synchronized populations were bleached and plated on NGM plates with OP50 at 20°C. Brood sizes and hatching rates were measured by counting eggs laid onto the plates and percent of viable embryos. Progenies of at least 15 animals were scored for each strain.

#### Microscopic imaging of P granule components

Images of GFP-tagged or mCherry-tagged P granule components were captured by an Axio Imager M2 microscope (Zeiss). Worms were constrained on RITE-ON glass slides (Beckton Dickinson) with 1 mM levamisole. Images were further processed and cropped by ImageJ programs. GFP:AID:GLH-1(DQAD) worms were kept on the plates with 500 mM indole-3-acetic acid (IAA, Afar aesar) for complete depletion of GLH-1 proteins until they grow to young adults. The animals were then transferred to regular plates and allow de novo expression of GLH-1(DQAD) over the time for imaging.

#### Preparation of small-RNA libraries and deep sequencing

Total RNAs were extracted from synchronous population of worms by Trizol (Sigma Alrich) and isopropanol precipitation. Small-RNAs were further purified by mir-Vana miRNA isolation kit (Thermo Scientific). Small-RNA cloning was carried out as previously described (Shen et al., 2018). In brief, homemade PIR-1 were used for trimming 5’ triphosphate of endogenous 22G-RNAs to monophosphate. The 3′ and 5′ adaptors were ligated using truncated ligase 2 (NEB) and ligase 1 (NEB) sequentially. cDNA were then produced by Superscript III reverse transcriptase (Thermo Scientific). cDNAs were amplified by Q5 polymerase (NEB) and barcodes sequences were added at this step. Amplified PCR products were subjected to native PAGE and size selection. High throughput sequencing was conducted using multiple Illumina platforms including High-Seq, Next-Seq and Nova-Seq. For sequencing of Argonaute-bound small-RNAs, Argonaute proteins/small-RNA complex were extracted from 50,000 synchronized worms and immunoprecipitated with anti-FLAG antibody (Sigma Aldrich) or GFP nanobody (Thermo Scientific). Small-RNAs were then extraced and purified with Trizol and ethanol precipitation. Small-RNA library preparation approach remains the same as described above.

#### Analysis of small-RNA sequencing

Overall sequencing data were analyzed using previously described pipeline (Gu et al., 2009; Shen et al., 2018). Raw sequencing data were demultiplexed and their 3′ adaptor trimmed and aligned to Wormbase WS262 and WS272 using Bowtie2 aligner. Unique mapped 22nt-long anti-sense reads were extracted for later analysis. Relative numbers of reads mapped to each gene were summed and normalized by total read counts of each sample (Reads per million, RPM). Genes enriched in IP WAGO-1 and CSR-1 were scored by limiting minimal 1 RPM and more than 2-fold enrichment over the levels of 22G-RNAs in the corresponding input data. Scatterplots were generated by python and matplotlib through comparing the normalized 22G-RNA read counts between two samples. Genes Genome browser view graphs were plotted as histograms (x axis, genomic coordinates; y axis, small-RNA density) by matplotlib. WAGO targets (1118 genes) and CSR-1 targets (4176 genes) were previously described (Claycomb et al., 2009; Gu et al., 2009). CSR-1 targets exhibiting ectopic 22G-RNAs were listed in Table S2.

#### Co-immunoprecipitation and western blotting

50,000 synchonized young adult worms were homogenized in lysis buffer (20 mM HEPES PH = 7.0, 250 mM Sodium Citrate, 0.5% Triton X-100, 0.1% Tween 20, 1 mM DTT, protease inhibitor) and the lysates were incubated with 2ul FLAG antibody (Clone M2, Sigma Aldrich) together with 30ul Protein G beads (Thermo Scientific) for FLAG-tagged WAGO-1 IP, or with GFP nanobody (Thermo Scientific) for GFP-tagged PRG-1 IP. Lysates were per-treated with RNase I (NEB) for 20 min at 20°C. After washing with lysis buffer, immunoprecipitates were eluted with 4X SDS sample buffer (BioRad) and were resolved by SDS-PAGE later transfer to PVDF membranes (BioRad). GLH-1 antibody (1:5000), GLH-4 antibody (1:2000), anti-FLAG HRP-conjugated antibody (Sigma, 1:5000) were used for probing the blots.

#### *In vitro* protein binding assay

MBP:WAGO-1, MBP:GFP, GLH-1(WT) and its variants were constructed onto pET-Duet1 vectors. FLAG-tagged GLH-1(WT), FLAG-tagged GLH-1 variants, MBP-tagged GFP and MBP-tagged WAGO-1 proteins were expressed in BL21 cells following 0.5 mM IPTG treatment for 18 h at 18 C. *E*. *coli* were collected and resuspend them in lysis buffer (50 mM Tris-HCl pH 8.0, 150 mM NaCl, 1 mM PMSF, 2 mM DTT). Bacterial homogenates were then sonicated and went through MBP-trap affinity column (GE health) by AKTA. With stringent washing with lysis buffer, protein complex were eluted with 10 mM Maltose (Sigma Aldrich). Resolve elutes by SDS-PAGE and transfer to PVDF membrane for western bloting. Anti-FLAG HRP antibody (Abcolonal, 1:5000) and anti-MBP antibody (MBL, 1:2500) were used for probing the blots.

#### Protein purification

Full-length FLAG-tagged GLH-1 was cloned into pET28a vector. The point mutations K391A and E500Q were introduced by site-directed mutagenesis (NEB). All constructs were expressed in ER2566 E.coli cells (WEIDI) at OD600 of 0.8 with 0.5 mM IPTG for 18 h at 18°C. Cells were resuspended in lysis buffer (50 mM Tris-HCl pH 8.0, 500 mM NaCl, 1 mM PMSF, 2 mM DTT) and was sonicated on the ice. Then the lysates were treated with 10 μL Ambion RNase Cocktail (Invitrogen) and 10 μL Micrococcal Nuclease (NEB) for removing bacterial RNAs that remain bound to the proteins. After high-speed centrifugation, the supernatant was loaded onto a column with 1 mL Anti-DYKDDDDK G1 Affinity Resin (GenScript) and the 1×FLAG-tagged proteins were affinity extracted. Then the resin was washed with 60 mL wash buffer (50 mM Tris-HCl pH8.0, 500 mM NaCl) and the proteins were eluted with 2 mL elution buffer (50 mM Tris-HCl pH8.0, 500 mM NaCl, 0.8 mg/mL FLAG peptide). After affinity purification, eluted samples were run on Superdex 200 (Sigma Aldrich) in gel filtration buffer (25 mM Tris-HCl pH8.0, 500 mM NaCl, 2 mM DTT) to separate aggregates with monomers according to their molecular weight difference (Figures S5F–S5H). All chromatography was conducted at 4°C. The abundance and purity of each collected fraction was validated by SDS-PAGE and Coomassie Blue staining. Finally, the collected monomers were combined and their concentration were measured by BCA Protein Assay Kit (Beyotime).

#### Electrophoretic mobility shift assay (EMSA)

RNA binding activities of purified GLH-1 WT, K391A and E500Q mutants were assessed by incubating 10 μM monomeric proteins and 2 μM 5′ end Cyanine-3 (Cy3) labeled RNAs (22 nt RNA 5′-Cy3-GUCAAAGAUAGCCUUGACCUUG-3′ for GLH-1 WT, mutants K391A and E500Q) in the presence and absence of 2 mM ATP for 30 min at RT in binding buffer (25 mM HEPES pH7.4, 150 mM NaCl, 2 mM MgCl2). The RNA-protein complexes were separated on native 8% polyacrylamide gel at 60 V in 13TBE buffer in cold room. Fluorescence signals were detected by BioRad imager.

#### *In vitro* helicase unwinding assays

Helicase unwinding assays were modified from previously described method (Huen et al., 2017). RNA duplex with 3’ extention were prepared by annealing 5′ Cyanine-3 labeled 11nt-long RNA oligo (5′-Cy3-AGCGCAGUACC-3′) with a 22nt-long RNA oligo (5′-GGUA-CUGCGCUUUUAUGACAUC-3′). 20 mM purified GLH-1 proteins (WT, K391A or DQAD) were incubated with 25 nM RNA duplex in helicase buffer (25 mM HEPES pH7.4, 150 mM NaCl, 2 mM MgCl_2_, 2 mM ATP) over the time. Unlabeled 11 nt RNA was added to a final concentration of 0.125 mM to prevent re-annealing of unwound products. The reactions were stopped by adding EDTA and the proteins were digested by adding Proteinase K (NEB) at 37°C for 20 min. RNA duplex and unwound products were subjected to native 15% polyacrylamide gel at 90 V in 1×TBE buffer at room termperture. Fluorensent Cy3 signals were detected by a BioRad imager.

#### CLIP sequencing to identify GLH-1 and GLH-4 binding RNAs

CLIP assays were modified from previously described CLIP methods (Chi et al., 2009; Nostrand et al., 2016). Two independent biological replicates were performed for each CLIP assay. Control libraries were also constructed from untagged strains expressing each *glh-1* allele and were used to account for unavoidable background RNA binding to the IP matrix. Independent synchronized populations of young adult animals were exposed to UV light and then subjected to extraction, partial RNase digestion (Thermo Scientific), and FLAG protein immunoprecipitation (Sigma Aldrich). Electrophoresis on a denaturing poly-acrylamide gel was then used to separate the proteins in the IP complex, followed by excision of a gel-slice corresponding in size to the GLH protein (and any associated cross-linked RNA fragments). RNA was then eluted from the gel slice and subjected to library construction and high-throughput sequencing.

#### Data analysis of CLIP

3′ adaptors were trimmed from raw data by Cutadapt and the remaining reads with at-least 15nt-long were kept for later analysis. Processed CLIP sequences were mapped to both genome and transcriptome of Wormbase WS262 using STAR genome aligner. Percentage of reads for each RNA class was calculated by dividing sum of mapped reads with total mapped read counts for the same sample. Relative aggregated CLIP reads mapped to each gene were calculated using HTseq and normalized by total read counts. GLH targets were scored by implementing filters for genes with at least two-fold enrichment of CLIP reads over untagged input data and minimal 5 RPM. To visualize CLIP binding over these target mRNA cateogires we generated a composite plot where CLIP reads from all genes in each group were plotted along a 5′ to 3′ metagene space, where each interval on the x axis represents 1% of the total mRNA length. Experimental and control target space were defined by mRNAs with over 2-fold reduction of 22G-RNAs in *glh-1*(*K391A*) worms and the transcripts with unaltered 22G-RNA levels. Distribution of CLIP-seq reads along the transcripts from 5′ to 3′ was plotted using deeptools.

### QUANTIFICATION AND STATISTICAL ANALYSIS

Sample sizes of each assay were described in figure legends, results section and STAR method section. Quantification of relative RNA affinity in Figure 5F and percentage of unwound RNA duplex in Figure 5G were conducted using ImageJ gel analysis tool. T test were used in Figures S1E and S1F to determine if there is a significant difference.

## Supplementary Material

1

## Figures and Tables

**Table:** KEY RESOURCES TABLE

REAGENT or RESOURCE	SOURCE	IDENTIFIER
Antibodies		

Anti-FLAG M2 antibody	Sigma-Aldrich	Cat# F1804; RRID:AB_262044
Anti-GLH-1 rabbit antibody	Gift from Karen Bennett lab	N/A
Anti-GLH-4 rabbit antibody	Gift from Karen Bennett lab	N/A
Anti-PRG-1 polyclonal antibody	Abcam	Cat# Ab15826; RRID:AB_777426
Anti-MBP mAb	MBL International	Cat# M091–3, RRID:AB_592157
Anti-GFP antibody	FUJIFILM Wako Shibayagi	Cat# 018–20463, RRID:AB_10659145
Anti-V5 Tag antibody	Abcam	Cat# ab27671, RRID:AB_471093
Anti-FLAG HRP-conjugated antibody	Sigma-Aldrich	Cat# A8592, RRID:AB_439702
Anti-DDDDK-Tag HRP-conjugated antibody	ABclonal	Cat# AE024, RRID:AB_2769864

Bacterial and virus strains		

*E*. *Coli*: OP50	CGC	Wormbase: OP50
*E*. *Coli*: HT115	CGC	Wormbase: HT115

Chemicals, peptides, and recombinant proteins		

Indole-3-acetec acid, IAA	Alfa Aesar	A10556
TRI Reagent	Sigma Aldrich	T9424
RNase If	NEB	M0243
MBP Resin	GE health	28,935,597
Maltose monohydrate	Sigma	6363–53-7
ATP, [γ-32P]- 6000Ci/mmol 10mCi/mL	Perkin Elmer	BLU002Z250UC
Dynabeads Protein G	Thermo	10003D
GFP nanobody	Chromotek	M-270

Critical commercial assays		

Pierce™ Silver Stain Kit	Thermo	24,612
*mir*Vana™ miRNA Isolation Kit	Thermo	AM1560

Deposited data		

Small-RNA seq and CLIP-seq data	This study	GEO: GSE195536 and GSE198101

Experimental models: Organisms/strains		

Strains used in this study	This paper	Table S3

Oligonucleotides		

Oligos used in this study	This paper	Table S4

Recombinant DNA		

pETDuet-1-his::MBP::TEV::WAGO-1	This paper	EZP0183
pETDuet-1-his::MBP::TEV::EGFP	This paper	EZP0184
pETDuet-1-FLAG::GLH-1	This paper	EZP0119
pETDuet-1-FLAG::GLH-1K391A	This paper	EZP0122
pETDuet-1-FLAG::GLH-1E550Q	This paper	EZP0243

Software and algorithms		

ImageJ	NIH	https://imagej.net/Welcome
Bowtie2	Bioconda	http://bowtie-bio.sourceforge.net/
STAR	Bioconda	https://github.com/alexdobin/STAR
Python3	Python.org	https://www.python.org
Pandas	Conda	https://pandas.pydata.org
Matplotlib	Conda	https://matplotlib.org
HTseq	PIP	https://htseq.readthedocs.io/
Deeptools	Bioconda	https://deeptools.readthedocs.io
BioRender	BioRender Team	https://biorender.com

## Highlights

- Argonautes recruit GLH proteins to mRNAs to promote transgenerational gene silencing
- GLH paralogs scaffold Argonautes and compete for target mRNA binding in nuage
- GLH proteins prevent misrouting of germline Argonaute pathways
- GLH-1 ATPase cycle promotes unwinding and regulates its binding to Argonaute
