# Supplementary material for: A family of C. elegans VASA homologs control Argonaute pathway specificity and promote transgenerational silencing
Source: Cell Rep. Author manuscript; Available in PMC 2023 Jan 31. (PMC9887883; doi:10.1016/j.celrep.2022.111265)
Supplement: 1 [file NIHMS1834786-supplement-1.pdf]

Cell Reports, Volume *40*

Supplemental information

A family of *C. elegans* VASA homologs control Argonaute pathway specificity and promote transgenerational silencing

Siyuan Dai, Xiaoyin Tang, Lili Li, Takao Ishidate, Ahmet R. Ozturk, Hao Chen, Altair L. Dude, Yong-Hong Yan, Meng-Qiu Dong, En-Zhi Shen, and Craig C. Mello

Figure S1

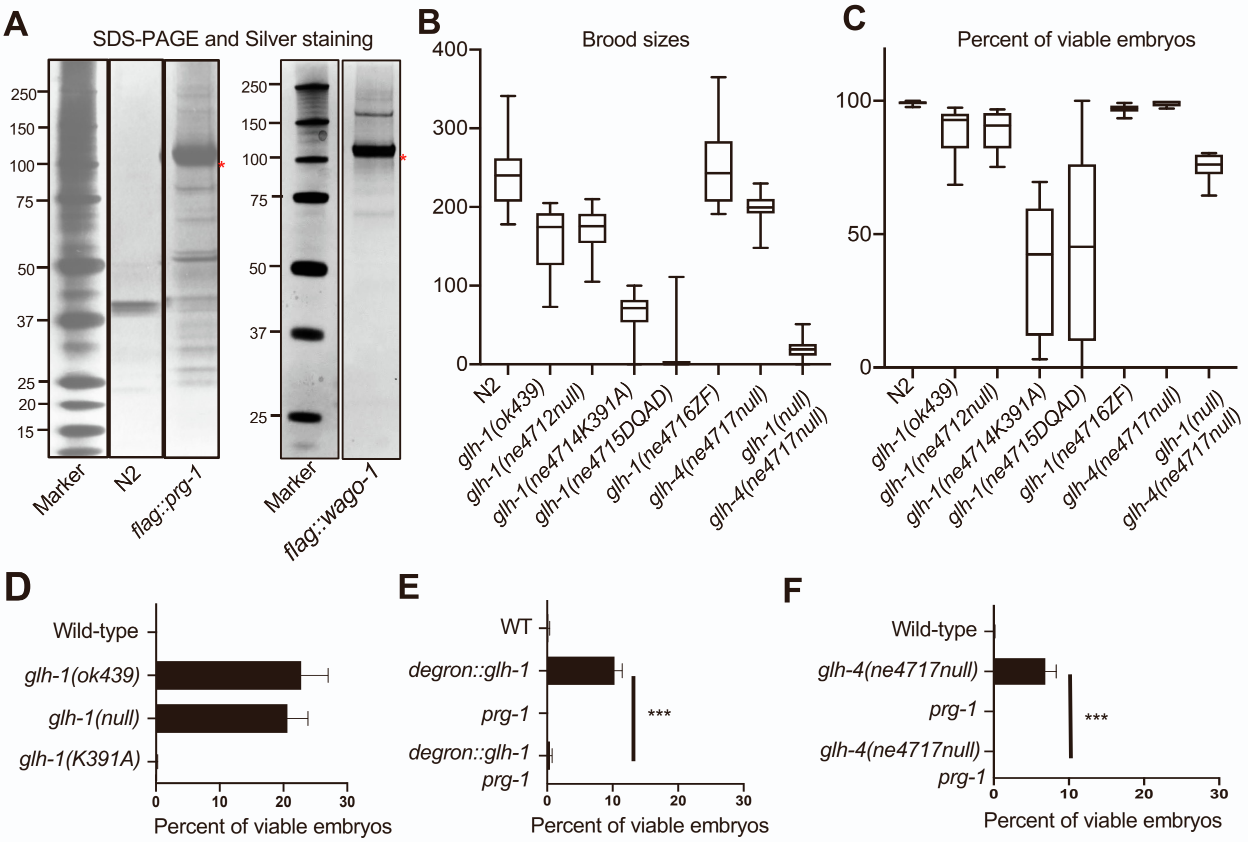

A
SDS-PAGE and Silver staining
250
150
100
75
50
37
25
20
15
Marker
N2
flag::prg-1
250
150
100
75
50
37
25
Marker
flag::wago-1
B
Brood sizes
400
300
200
100
0
N2
glh-1(ok439)
glh-1(ne4712null)
glh-1(ne4714K391A)
glh-1(ne4715DQAD)
glh-1(ne4716ZF)
glh-4(ne4717null)
glh-1(null)
glh-4(ne4717null)
C
Percent of viable embryos
100
50
0
N2
glh-1(ok439)
glh-1(ne4712null)
glh-1(ne4714K391A)
glh-1(ne4715DQAD)
glh-1(ne4716ZF)
glh-4(ne4717null)
glh-1(null)
glh-4(ne4717null)
D
Wild-type
glh-1(ok439)
glh-1(null)
glh-1(K391A)
0
10
20
30
Percent of viable embryos
E
WT
degron::glh-1
prg-1
degron::glh-1
prg-1
***
0
10
20
30
Percent of viable embryos
F
Wild-type
glh-4(ne4717null)
prg-1
glh-4(ne4717null)
prg-1
***
0
10
20
30
Percent of viable embryos

Figure S1: Identification of PRG-1 and WAGO-1 associated proteins. Fertility of glh-1 mutant strains. *glh* mutants exhibited cold-sensitive phenotypes.

(A): Purification of PRG-1 and WAGO-1 proteins: 3XFLAG-tagged PRG-1 and WAGO-1 was immune-precipitated and resolved by SDS-PAGE. Proteins were visualized by silver staining. Immuno-precipitated PRG-1 and WAGO-1 were indicated as red asterisks.

(B, C): Brood sizes (B) and precent of viable embryos (C) for each indicated strain. Error bars indicate standard deviation from the mean.

(D) *pos-1* RNAi assay for wild-type, *glh-1(ok439), glh-1(null) and glh-1(K391A)* animals. Worms are fed with *pos-1* RNAi from L1 stage at 15°C and percentage of their viable offspring was monitored. More than 300 eggs were scored for each strain.

(E, F) *pos-1* RNAi assay for auxin-depleted GLH-1 or *glh-4* null animals double with *prg-1* mutant. Worms were fed with *pos-1* RNAi on plates containing both auxin and IPTG at 15°C. Over 300 eggs were scored. ***, $P<0.001$ (t-test)

Figure S2

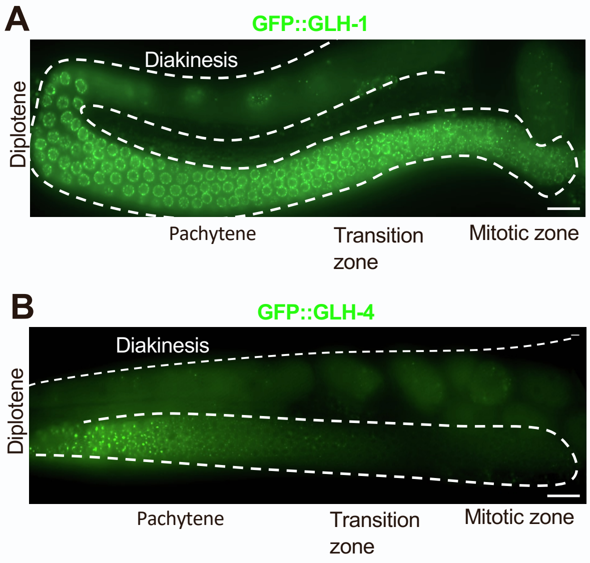
A
GFP::GLH-1
Diakinesis
Diplotene
Pachytene
Transition zone
Mitotic zone
B
GFP::GLH-4
Diakinesis
Diplotene
Pachytene
Transition zone
Mitotic zone

Figure S2: GLH-1 and GLH-4 exhibit distinct expression patterns.

(A, B) Fluorescence microscopy showing GLH-1 and GLH-4 localization within one side of worm gonad. Scale bar, 20um.

Figure S3

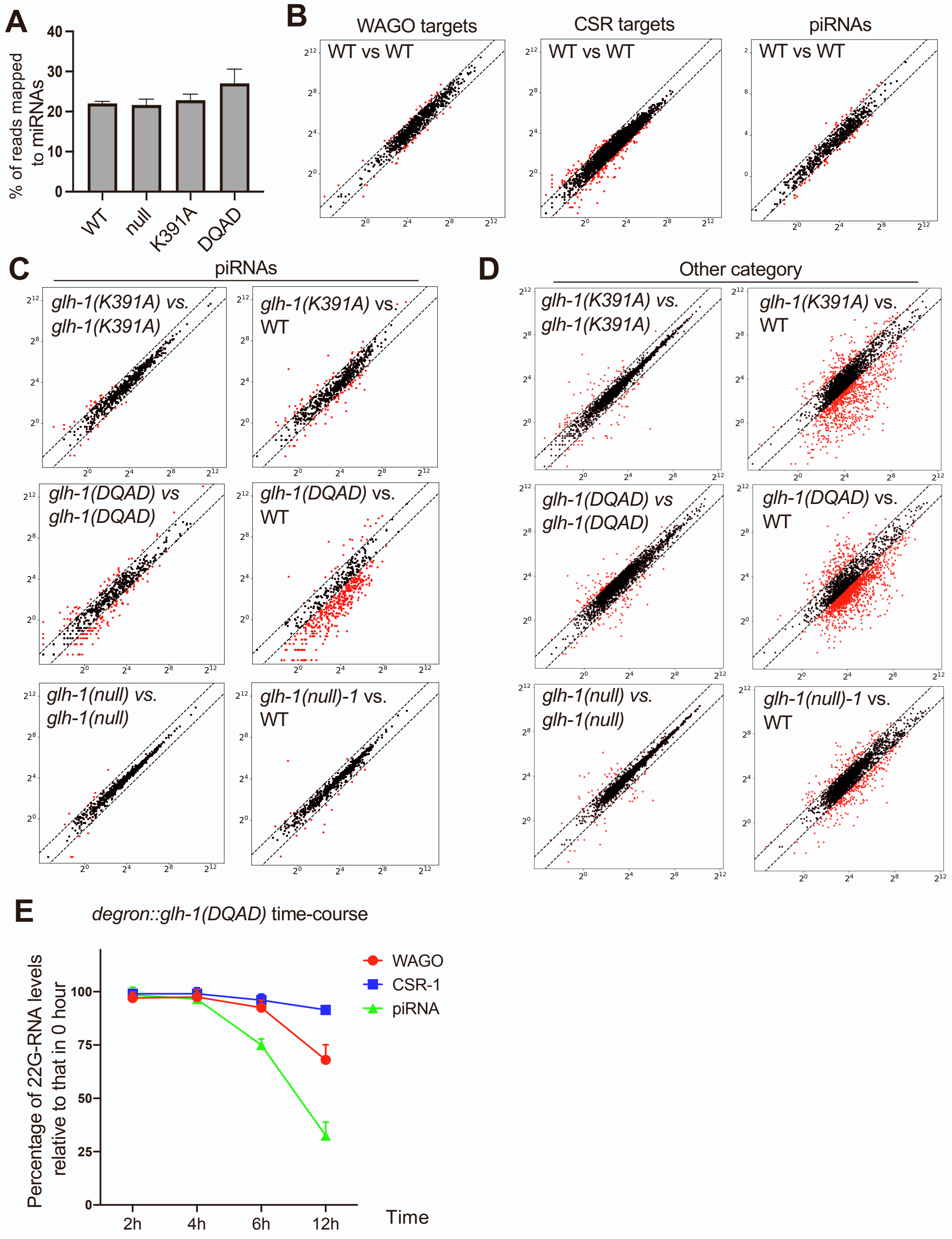
A
% of reads mapped to miRNAs
40
30
20
10
0
WT
null
K391A
DQAD
B
WAGO targets
CSR targets
piRNAs
WT vs WT
WT vs WT
WT vs WT
C
piRNAs
glh-1(K391A) vs. glh-1(K391A)
glh-1(K391A) vs. WT
glh-1(DQAD) vs glh-1(DQAD)
glh-1(DQAD) vs. WT
glh-1(null) vs. glh-1(null)
glh-1(null)-1 vs. WT
D
Other category
glh-1(K391A) vs. glh-1(K391A)
glh-1(K391A) vs. WT
glh-1(DQAD) vs glh-1(DQAD)
glh-1(DQAD) vs. WT
glh-1(null) vs. glh-1(null)
glh-1(null)-1 vs. WT
E
degron::glh-1(DQAD) time-course
Percentage of 22G-RNA levels relative to that in 0 hour
100
75
50
25
0
2h
4h
6h
12h
Time
WAGO
CSR-1
piRNA

Figure S3: Abundance of various classes of small RNAs in *glh-1* mutant animals.

(A) Percent of reads mapped to annotated miRNAs out of total small-RNA sequencing reads.

(B) Scatterplots comparing the numbers of small-RNAs for WAGO targets (1118 genes), CSR-1 targets (3206 genes) and abundant piRNAs (621 genes) between two biological replicates cloned from wild-type worms. Dotted lines indicate two-fold threshold.

(C) Scatterplots comparing the numbers of abundant piRNAs between two biological replicates and cloned from wild-type, *glh-1(K391A)*, *glh-1(DQAD)* and *glh-1(null)* animals. Dotted lines indicate two-fold threshold.

(D) Scatterplot comparing the numbers of small-RNAs for other categories (Excluding WAGO and CSR-1 targets) between two biological replicates and cloned from wild-type, *glh-1(K391A)*, *glh-1(DQAD)* and *glh-1(null)* animals. Dotted lines indicate two-fold threshold.

(E) Relative abundance of different classes of small-RNAs (22G-RNAs on WAGO targets, 22G-RNAs on CSR-1 targets and piRNAs) over the time with *de novo* expression of GLH-1(DQAD).

Figure S4

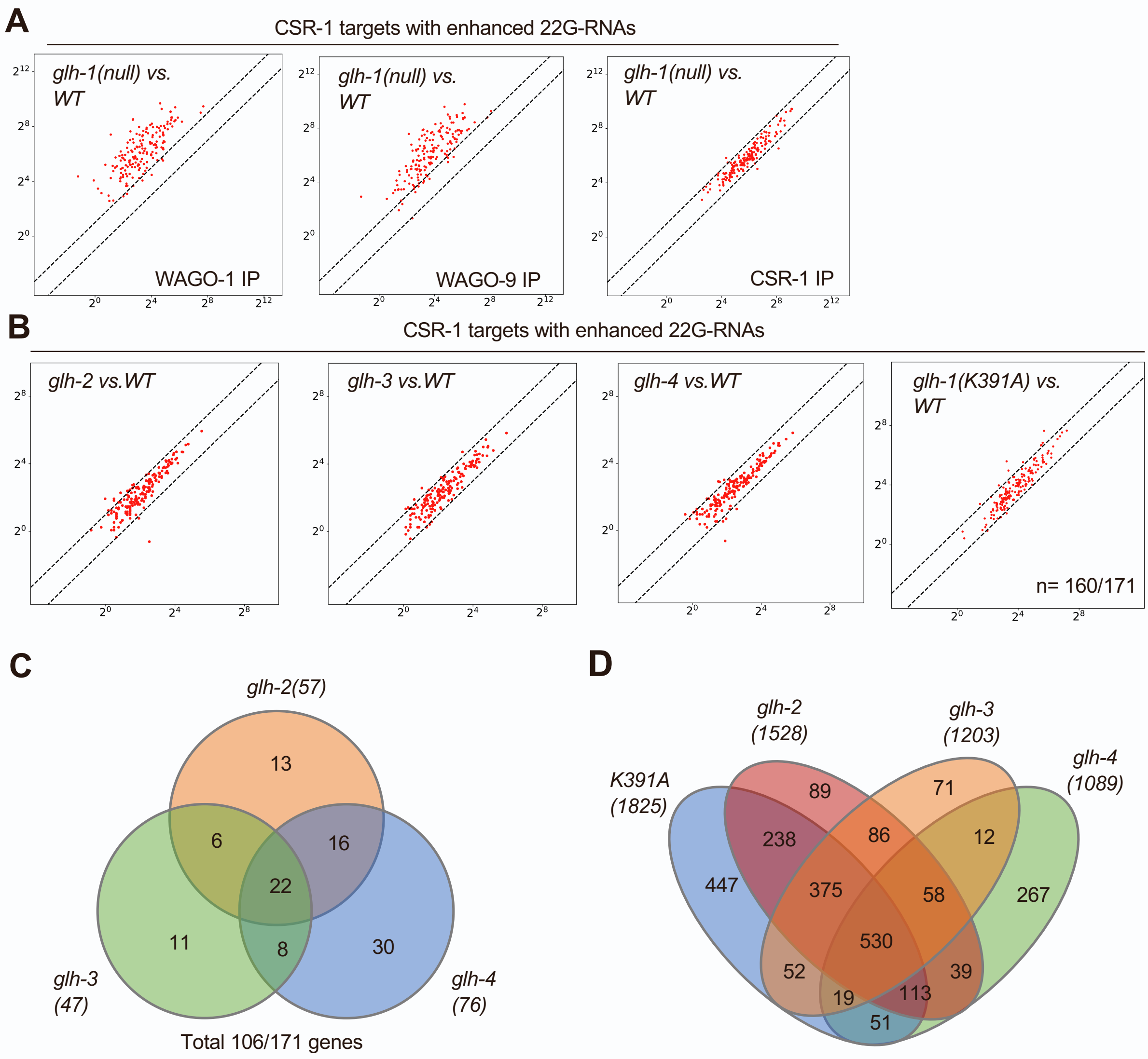
A
CSR-1 targets with enhanced 22G-RNAs
glh-1(null) vs. WT
WAGO-1 IP
glh-1(null) vs. WT
WAGO-9 IP
glh-1(null) vs. WT
CSR-1 IP
B
CSR-1 targets with enhanced 22G-RNAs
glh-2 vs.WT
glh-3 vs.WT
glh-4 vs.WT
glh-1(K391A) vs. WT
n= 160/171
C
glh-2(57)
13
6
16
22
11
8
30
glh-3 (47)
glh-4 (76)
Total 106/171 genes
D
glh-2 (1528)
glh-3 (1203)
glh-4 (1089)
K391A (1825)
89
71
238
86
12
447
375
58
267
530
52
39
19
113
51

Figure S4: Ectopic WAGO 22G-RNAs in *glh-1* mutant are suppressed in the mutants of *glh* paralogs.

(A): 171 CSR-1 targets with enhanced WAGO 22G-RNAs. Scatterplot depicting 22G-RNAs cloned via WAGO-1, WAGO-9 and CSR-1 IP from WT and *glh-1(null)* animals on 171 targets. Dotted lines indicate two-fold threshold.

(B): Scatterplots showing 22G-RNAs cloned from WT and *glh-2, glh-3, glh-4* and *glh-1(K391A)* animals. Dotted lines indicate two-fold threshold.

(C): Venn diagram depicting the numbers of CSR-1 targets with marked reduction of ectopic 22G-RNAs in *glh-1* double mutants. *degron::glh-2;glh-1* (orange), *degron::glh-3;glh-1* (green), *degron::glh-4;glh-1* (blue).

(D): Venn diagram depicting the numbers of genes (all) with more than 2-fold reduced 22G-RNAs in *glh-1(K391A)* (blue), *degron::glh-2;glh-1* (red), *degron::glh-3;glh-1* (orange) and *degron::glh-4;glh-1* (green) animals.

Figure S5

A

Proteins	# peptides by IP-MS		
	Control	FLAG::GLH-1 IP	FLAG::GLH-1 (K391A)
GLH-1	2	2706	2346
GLH-2	0	1116	1149
GLH-3	0	115	110
GLH-4	0	24	0
PRG-1	0	107	153
WAGO-1	0	91	164
WAGO-3	0	42	75
WAGO-4	0	82	45
WAGO-5	0	16	15
CSR-1	0	9	10
PAB-1	10	60	128
DEPS-1	0	45	49
ZNFX-1	0	67	83
LAF-1	0	12	0
VBH-1	0	10	0
CGH-1	2	23	0

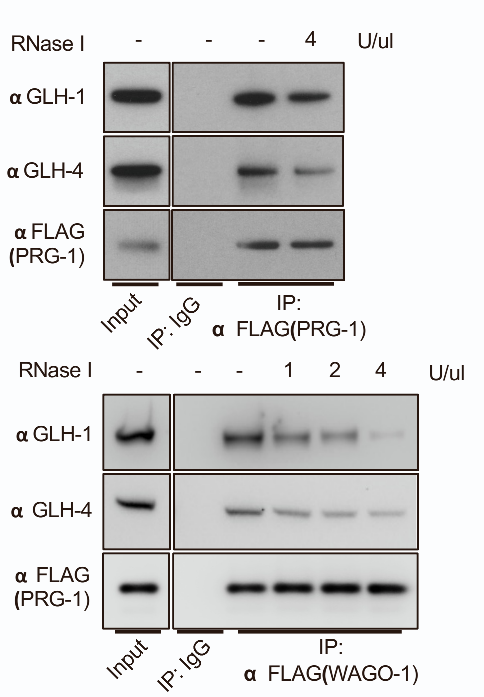

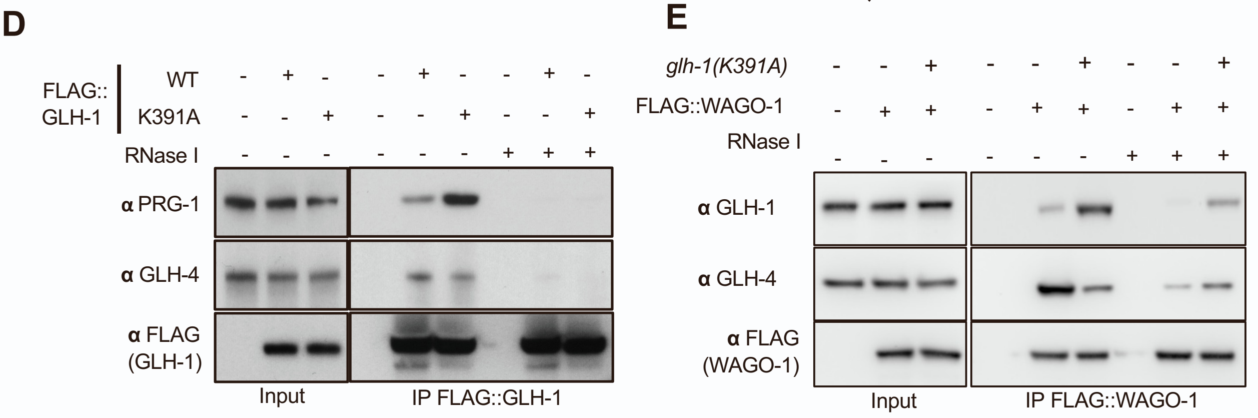

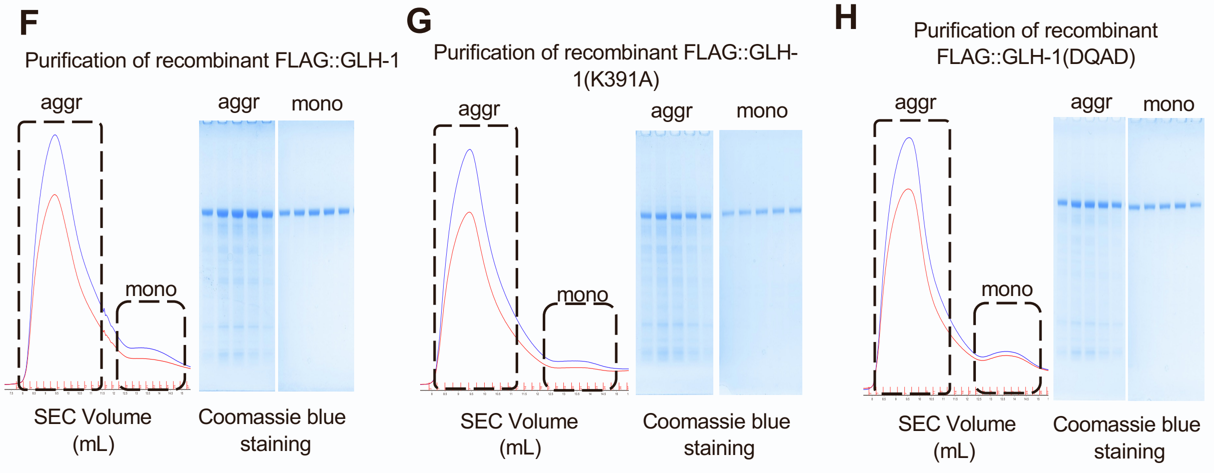

Figure S5: K391A enhances GLH-1's binding to PRG-1 and WAGO-1 Argonautes.

(A): Analysis of IP-MS results by immuno-precipitating GLH-1 WT and GLH-1 K391A protein. Peptide spectra counts for selected co-factors were shown in the table.

(B, C): Replication of Co-IP and WB experiments for Fig 5A and Fig 5B.

(D): A reciprocal Co-IP experiment showing interactions between GLH-1 and PRG-1 or GLH-4 in WT or mutant animals expressing GLH-1(K391A) protein. Presence (+) or absence (-) of RNase I were indicated.

(E): A repeat of Co-IP and WB experiment for Figure 5D. Blots depict the interactions between WAGO-1 and GLH-1 WT or GLH-1 K391A. Presence (+) or absence (-) of RNase I were indicated.

(F, G, H): 280/260 UV absorbance graph was shown to indicate the amounts of proteins of different molecular weights separated by size exclusion chromatography. Protein aggregates (aggr) and monomers (mono) were outlined in the graphs. The collected fractions were resolved by SDS-PAGE and stained with Coomassie Blue.

Figure S6

A

Breakdown of mapped CLIP reads

Samples	mRNA	piRNA	Anti-sense	rRNA	miRNA	tRNA	other
GLH-1	88%	6%	<1%	4%	<1%	<1%	2%
GLH-1;*prg-1*	89%	<1%	<1%	5%	<1%	<1%	4%
GLH-1;*rde-3*	87%	4%	<1%	4%	<1%	<1%	5%
GLH-4	53%	25%	<1%	18%	<1%	<1%	4%
GLH-4 ;*glh-1*	54%	21%	<1%	16%	<1%	<1%	7%
K391A	88%	3%	<1%	2%	<1%	<1%	6%
DQAD 0h	42%	<1%	<1%	43%	<1%	<1%	14%
DQAD 2h	79%	<1%	<1%	6%	<1%	<1%	15%
DQAD 4h	49%	<1%	<1%	35%	<1%	<1%	15%

B

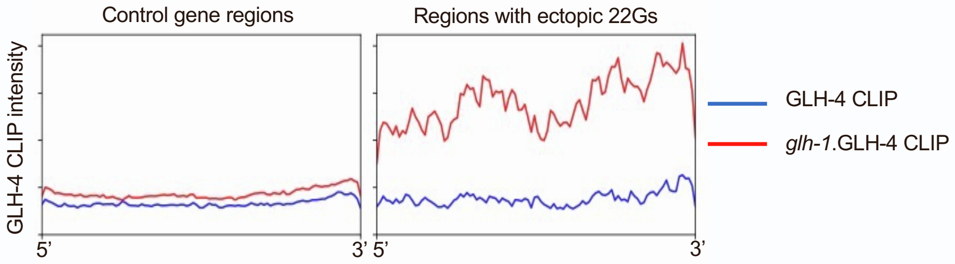

Figure S6: GLH-1-associated RNAs are predominantly mRNAs. Enhanced binding of GLH-4 to mRNAs in *glh-1(null)* mutant correlate with ectopic WAGO 22G-RNAs.

(A): Table depicting the fractions of CLIP reads mapped to each RNA class (mRNAs, piRNAs, ribosomal RNAs, micro RNAs, transfer RNAs and other categories).

(B): Metagene analysis of GLH-4 CLIP in WT and *glh-1(null)* mutants over control gene regions and gene regions with ectopic 22G-RNAs. Y-axis indicates relative GLH-4 CLIP intensity.

Table S1, IP-Mass Spectrometry results, Related to Figure 1.

PRG-1 IP-MS:

Genge	Gene ID	Description
wago-1*	R06C7.1	Argonaute
cpz-1	F32B5.8	Cysteine proteinase
glh-4*	T12F5.3	RNA helicase
pab-1*	Y106G6H.2	Poly-adenylate binding protein
C37A2.7*	C37A2.7	ribosomal protein lateral stalk subunit P2
rla-1*	Y37E3.7	ribosomal protein lateral stalk subunit P1
rps-21*	F37C12.11	Ribosomal protein small subunit
glh-1*	T21G5.3	RNA helicase
hsp-6*	C37H5.8	Heat shock protein
rps-12	F54E7.2	Ribosomal protein small unit
rpl-36*	F37C12.4	Ribosomal protein large unit
aldo-1	T05D4.1	Fructose-bisphosphate aldolase class-I
pck-2	R11A5.4	phosphoenolpyruvate carboxykinase
hda-10	Y51H1A.5	histone deacetylase
rpl-3*	F13B10.2	Ribosomal protein large unit
nol-58*	W01B11.3	ribonucleoprotein
pdi-3	H06O01.1	protein disulphide isomerase
wago-4*	F58G1.1	Argonaute
rpl-12*	JC8.3	Ribosomal protein large unit
rps-11*	F40F11.1	Ribosomal protein small unit
pdi-1	C14B1.1	protein disulfide isomerase
rbm-3.2	R09B3.3	RNA binding motif protein 3
pars-1	T20H4.3	glutamyl-prolyl-tRNA synthetase
tbb-1	K01G5.7	Prolyl Amino-acyl tRNA Synthetase
hsp-4*	F43E2.8	heat shock protein
vgln-1	C08H9.2	high density lipoprotein binding protein
pdi-6	B0403.4	protein disulfide isomerase
sip-1	F43D9.4	heat shock protein
ubl-1*	H06I04.4	ubiquitin-Like protein
gst-36	R07B1.4	glutathione peroxidase
rpl-16	M01F1.2	Ribosomal protein large unit
rla-0	K01G5.7	ribosomal protein lateral stalk subunit P0

WAGO-1 IP-MS:

Gene	Gene ID	Description
F46G10.1	F46G10.1	ortholog of human KCTD18
wago-5	ZK1248.7	Argonaute
pab-1	Y106G6H.2	Poly-adenylate binding protein
pgl-1	ZK381.4	P-granule component
fib-1	T01C3.7	ortholog of human FBL (fibrillarin)
rps-2	C49H3.11	ribosomal protein small unit
eef-1A.2	R03G5.1	elongation factor
rps-4	Y43B11AR.4	ribosomal protein small unit
T23E7.2	T23E7.2	ortholog of human FAM186A

rps-4	C23G10.3	ribosomal protein small unit
glh-4	T12F5.3	RNA helicase
ddx-17	F58E10.3	RNA helicase
daf-21	C47E8.5	heat shock protein
deps-1	Y65B4BL.2	P-granule component
rps-8	F42C5.8	ribosomal protein small unit
nol-58	W01B11.3	ribonucleoprotein
rps-11	F40F11.1	ribosomal protein small unit
hrpf-1	F58D5.1	heterogeneous nuclear ribonucleoprotein R
pab-2	F18H3.3	Poly-adenylate binding protein
nol-56	K07C5.4	ribonucleoprotein
C27B7.5	C27B7.5	nucleic acid binding activity and zinc ion binding activity
pgl-3	C18G1.4	P-granule component
ppw-2/wago-3	Y110A7A.18	Argonaute
rpl-4	B0041.4	ribosomal protein large unit
rps-14	F37C12.9	ribosomal protein small unit
rla-1	Y37E3.7	ribosomal protein lateral stalk subunit P1
rps-1	F56F3.5	ribosomal protein small unit
unc-54	F11C3.3	myosin heavy chain
lfi-1	ZC8.4	ciliary rootlet coiled-coil, rootletin
rpl-21	C14B9.7	ribosomal protein large unit
rps-5	T05E11.1	ribosomal protein small unit
rpl-23	B0336.10	ribosomal protein large unit
rpl-10	F10B5.1	ribosomal protein large unit
vit-2	C42D8.2	vitellogenin structural genes
car-1	Y18D10A.17	mRNA processing body assembly factor
prg-1	D2030.6	Argonaute
Y111B2A.3	Y111B2A.3	unannotated
atx-2	D2045.1	ataxin 2 like
atp-1	H28O16.1	TP synthase F1 subunit alpha
rps-16	T01C3.6	ribosomal protein small unit
laf-1	Y71H2AM.19	RNA helicase
rpl-3	F13B10.2	ribosomal protein large unit
perm-2	C44B12.1	PERMeable eggshell
rpl-20	E04A4.8	ribosomal protein large unit
C37A2.7	C37A2.7	ribosomal protein lateral stalk subunit P2
rpl-18	Y57G11C.16	ribosomal protein large unit
rpl-11.1	T22F3.4	ribosomal protein large unit
rps-6	Y71A12B.1	ribosomal protein small unit
rpl-18	Y45F10D.12	ribosomal protein large unit
rpl-36	F37C12.4	ribosomal protein large unit
vbh-1	Y54E10A.9	RNA helicase
wago-4	F58G1.1	Argonaute
gtbp-1	K08F4.2	G3BP stress granule assembly factor 1
rps-21	F37C12.11	ribosomal protein small unit
ima-3	F32E10.4	importin aphla
Y42H9AR.1	Y42H9AR.1	golgi reassembly stacking protein
K01G5.5	K01G5.5	dyskerin pseudouridine synthase

fbxa-81	T24C2.4	F-box protein class A
rpl-17	Y48G8AL.8	ribosomal protein large unit
dim-1	C18A11.7	myopalladin
nono-1	F25B5.7	non-POU domain containing octamer binding
K07H8.10	K07H8.10	RNA binding protein
glh-1	T21G5.3	RNA helicase
rps-23	F28D1.7	ribosomal protein small unit
rpl-9	R13A5.8	ribosomal protein large unit
cpf-1	F28C6.3	cleavage and polyadenylation factor
rpl-25.1	F55D10.2	ribosomal protein large unit
rps-27	F56E10.4	ribosomal protein small unit
hsp-4	F43E2.8	heat shock protein
rpl-7A	Y24D9A.4	ribosomal protein large unit
abcf-2	T27E9.7	ABC transporter, class F
sqd-1	Y73B6BL.6	heterogeneous nuclear ribonucleoprotein A/B
pqn-59	R119.4	ubiquitin associated protein 2
rps-15	F36A2.6	ribosomal protein small unit
vig-1	F56D12.5	Drosophila Vasa Intronic Gene
R148.3	R148.3	unannotated
rpl-31	W09C5.6	ribosomal protein large unit
gld-1	T23G11.3	KH domain containing RNA binding
hsp-6	C37H5.8	heat shock protein
hrpu-2	Y71G10AL.1	Heterogeneous nuclear RibonucleoProtein U
idha-1	F43G9.1	socitrate dehydrogenase
vit-3	F59D8.1	vitellogenin structural genes
mek-5	E02D9.1	KH RNA binding domain protein
rpl-43	Y48B6A.2	ribosomal protein large unit
T28C6.7	T28C6.7	unannotated
cey-2	F46F11.2	Y-box binding protein
rpl-34	C42C1.14	ribosomal protein large unit
lrp-1	F29D11.1	LDL receptor related protein
cbd-1	H02I12.1	Chitin-Binding Domain protein
tiar-1	C18A3.5	TIA1 cytotoxic granule associated RNA binding protein
catp-7	Y59H11AR.2	Cation transporting ATPase
rps-13	C16A3.9	ribosomal protein small unit
rpl-12	JC8.3	ribosomal protein large unit
perm-4	C44B12.5	permeable eggshell
xrn-2	Y48B6A.3	5'-3' exoribonuclease
rps-22	F53A3.3	ribosomal protein small unit
rps-20	Y105E8A.16	ribosomal protein small unit
Y37E3.8a	Y37E3.8	ribosomal protein large unit
rpl-30	Y106G6H.3	ribosomal protein large unit
ant-1.4	T01B11.4	adenine nucleotide translocator
suca-1	F47B10.1	succinate-CoA ligase
fbxa-101	F08A8.7	F-box protein class A
vit-5	C04F6.1	vitellogenin structural genes
golg-2	F33G12.5	GOLGi associated coiled-coil protein
hsp-25	C09B8.6	heat shock protein

rps-10	D1007.6	ribosomal protein small unit
rpl-19	C09D4.5	ribosomal protein large unit
rps-25	K02B2.5	ribosomal protein small unit
ngp-1	T19A6.2	Nuclear/nucleolar GTP-binding Protein
rpl-28	R11D1.8	ribosomal protein large unit
tps-2	F19H8.1	Trehalose 6-phosphate synthase
elks-1	F42A6.9	ELKS/RAB6-interacting/CAST family member
mpz-1	C52A11.4	Multiple PDZ domain protein
grdn-1	Y51A2D.15	coiled-coil domain containing protein
B0261.7	B0261.7	unannotated
imb-3	C53D5.6	importin beta
ifa-3	F52E10.5	Intermediate Filament
eat-6	B0365.3	ATPase Na+/K+ transporting subunit alpha
ubl-1	H06I04.4	ubiquitin associated protein
phb-2	T24H7.1	Prohibitin
rpl-24.1	D1007.12	ribosomal protein large unit
rpl-32	T24B8.1a	ribosomal protein large unit
rbm-39	Y55F3AM.3	RNA binding motif protein 39
mua-6	W10G6.3	lamin B2
pph-5	Y39B6A.2	protein phosphatase
lev-11	Y105E8B.1	tropomyosin

Table S2. 171 CSR-1 targets exhibiting ectopic 22G-RNAs, Related to Figure 4.

B0205.1, B0273.3, B0414.3, B0491.6, C03H5.3, C04A2.3, C04H5.6, C07H6.2, C09G12.8, C14B1.12, C14C11.2, C14C11.6, C15H11.5, C16C10.1, C17F4.5, C18E3.2, C26F1.3, C27B7.5, C27D9.1, C28D4.3, C29A12.1, C32D5.5, C33A12.3, C35D10.7, C37A2.4, C47B2.5, C47D12.6, C48A7.2, C49H3.3, D2005.4, F08G5.1, F10E7.5, F10E9.11, F17C11.8, F22B3.4, F22G12.4, F25D7.4, F26F4.11, F26G5.9, F26H11.2, F28F8.5, F28F8.6, F32A7.5, F32D1.1, F33H2.1, F33H2.3, F34D10.4, F35D11.5, F36H1.4, F37C12.13, F40F8.9, F40F9.7, F42A9.6, F42H11.2, F43D9.4, F52C6.11, F53F1.2, F53F8.5, F54D10.5, F55A3.3, F55C7.7, F55F8.6, F55G1.8, F57C7.1, F57C9.1, F58G1.1, F59A3.6, F59C6.4, F59E12.4, F59E12.5, H02I12.8, H06H21.11, H20J04.6, K03B4.1, K04F10.7, K05C4.2, K07C5.3, K08F11.5, K09H9.6, K10C3.2, K10C3.6, M04F3.1, R02F2.1, R05D11.6, R06C7.4, R07C3.2, R07E5.10, R107.5, R11A5.2, R12E2.12, R144.10, T03F6.2, T05B9.1, T06C12.4, T08D2.7, T10B11.8, T10C6.7, T10G3.5, T12C9.7, T16H12.11, T21B10.4, T23B5.1, T24H10.1, T24H10.3, T25E12.5, T26A8.4, T26C12.1, T28A8.4, T28A8.5, W01G7.4, W02B9.1, W02D3.11, W02D9.3, W02G9.3, W03F8.4, W03G9.2, W04A8.1, Y102A5C.1, Y111B2A.1, Y116A8C.12, Y116A8C.16, Y17G7B.15, Y24D9A.2, Y24D9A.8, Y37E11B.3, Y38A10A.7, Y38F2AR.3, Y39A1A.1, Y39E4B.2, Y39G10AR.13, Y39G10AR.14, Y41C4A.10, Y41D4B.13, Y41D4B.4, Y41E3.1, Y44E3A.6, Y45F3A.2, Y45G5AM.2, Y46G5A.1, Y46G5A.2, Y48A6B.3, Y48B6A.11, Y48B6A.3, Y48G1BL.1, Y48G1BM.1, Y48G1C.1, Y49E10.2, Y49F6B.9, Y51A2D.7, Y51H4A.15, Y53C12B.1, Y53C12B.2, Y54E5B.3, Y55B1AL.3, Y57A10A.19, Y57A10A.5, Y59A8B.13, Y60A3A.12, Y63D3A.5, Y66D12A.10, Y66D12A.9, Y69A2AR.6, Y71F9AL.18, Y73B3B.1, Y77E11A.1, Y94H6A.3 , ZC155.3, ZC317.7, ZK418.5, ZK637.5, ZK652.6

Table S3, *C. elegans* strains, Related to STAR methods:

Strain name:	Genotype	Methods/reference
N2	Wild-type	CGC
WM604	*prg-1(ne4484[flag::tev::prg-1]) I*	CRISPR. Ishidate et al., 2018
WM603	*wago-1(ne4585[3Xflag::TEV::SNAP::WAGO-1])I*	CRISPR Ishidate et al., 2018
WM531	*neSi12[cdk-1::gfp; Cbr-unc-119(+)] II; unc-119(ed9) III*; *[21ux-1 (anti-gfp) (ne4561)] X*	MosSCI, CRISPR, Shen et al., 2018
WM241	*neSi11[gfp::cdk-1(RNAe), cb-unc-119(+)] II; unc-119(ed3) III*	MosSCI, Shirayama et al., 2012
WM791	*glh-1(ok439) I; neSi11 [gfp::cdk-1(RNAe), cb-unc-119(+)] II; unc-119(ed3) III*	Cross
WM792	*glh-1(ne4712[null]) I; neSi11 [gfp::cdk-1(RNAe), cb-unc-119(+)] II; unc-119(ed3) III*	CRISPR
WM793	*glh-1(ne4714[K391A]) I*; *neSi11 [gfp::cdk-1(RNAe), cb-unc-119(+)] II; unc-119(ed3) III*	CRISPR
WM794	*glh-1(ne4715[DQAD]) I*; *neSi11 [gfp::cdk-1(RNAe), cb-unc-119(+)] II; unc-119(ed3) III*	CRISPR
WM795	*glh-1(ne4716[Zinc finger deletion]) I; neSi11 [gfp::cdk-1(RNAe), cb-unc-119(+)] II; unc-119(ed3) III*	CRSIPR
WM796	*glh-4(ne4717[null]) I; neSi11 [gfp::cdk-1(RNAe), cb-unc-119(+)] II; unc-119(ed3) III*	CSRIPR
WM242	*neSi12[cdk-1::gfp, cb-unc-119(+)] II; unc-119(ed3) III.*	MosSCI
EZS314	*glh-1(ne4891[null]) I; neSi12 [cdk-1::gfp, cb-unc-119(+)] II; unc-119(ed3) III*	CSRIPR
EZS176	*glh-1(ne4892[K391A]) I; neSi12 [cdk-1::gfp, cb-unc-119(+)] II; unc-119(ed3) III*	CRSIPR
WM825	*glh-1(ne4919[degron::glh-1]) I; neSi81(Psun-1::tir-1::mRuby::eft 3'utr) IV*	CRSIPR
WM826	*glh-1(ne4919[degron::glh-1]) I; prg-1(ne4920); neSi81(Psun-1::tir-1::mRuby::eft 3'utr) IV*	CRSIPR
WM161	*prg-1(tm872) I;*	ENU
WM797	*glh-4(ne4717[null]) prg-1(tm872) I*	Cross

WM704	*glh-1(ne4816[gfp::glh-1]) I*	CRISPR
WM798	*glh-1(ne4895[gfp::glh-1, K391A]) I*	CRISPR
WM799	*glh-1(ne4896[gfp::glh-1, DQAD]) I*	CRISPR
WM801	*glh-1(ne4898[null]) mcherry::pgl-1 I*	CRSIPR
WM802	*glh-1(ne4899[gfp::glh-1, K391A]) mcherry::pgl-1 I*	CRSIPR
WM803	*glh-1(ne4900[gfp::glh-1, DQAD]) mcherry::pgl-1 I*	CRSIPR
WM820	*prg-1(ne4915[gfp::prg-1]) I; csr-1(ne4515[mcherry::csr-1]) IV;*	CRSIPR
WM821	*glh-1(ne4916[null]) prg-1(ne4915[gfp::prg-1]) I; csr-1(ne4515[mcherry::csr-1]) IV;*	CRSIPR
WM822	*glh-1(ne4917[gfp::glh-1, K391A]) prg-1(ne4915[gfp::prg-1]) I; csr-1(ne4515[mcherry::csr-1]) IV;*	CRSIPR
WM823	*glh-1(ne4918[gfp::glh-1, DQAD]) prg-1(ne4915[gfp::prg-1]) I; csr-1(ne4515[mcherry::csr-1]) IV;*	CRSIPR
WM607	*wago-1(ne[gfp::wago-1]) I*	CRISPR, Ghanta et al., 2019
WM811	*glh-1(ne4908[null]) gfp::wago-1 I*	CRSIPR
WM813	*glh-1(ne4910[gfp::glh-1, K391A]) gfp::wago-1 I*	CRSIPR
WM815	*glh-1(ne4912[gfp::glh-1, DQAD]) gfp::wago-1 I*	CRSIPR
WM800	*glh-4(ne4897[gfp::glh-4]) I*	CRISPR
WM812	*glh-1(ne4909[null]) glh-4(ne4897[gfp::glh-4]) I*	CRSIPR
WM814	*glh-1(ne4911[gfp::glh-1, K391A]) glh-4(ne4897[gfp::glh-4]) I*	CRSIPR
WM816	*glh-1(ne4913[gfp::glh-1, DQAD]) glh-4(ne4897[gfp::glh-4]) I*	CRSIPR
WM824	*glh-1(ne4894[gfp::degron::glh-1, DQAD]) I; neSi81(Psun-1::tir-1::mRuby::eft 3'utr) IV*	CRSIPR
WM521	*wago-9(ne4336[flag::tev::wago-9]) III;*	CRSIPR
WM804	*glh-1(ne4712[null]) I; wago-9(ne[flag::wago-9]) III;*	Cross
WM806	*glh-1(ne4712[null]) I; csr-1(ne4520[flag::csr-1]) IV*	Cross
WM805	*glh-1(ne4901[null]) wago-1(ne4585[flag::wago-1]) I;*	CRSIPR

EZS292	*glh-1(ne4712[null]) prg-1(ne4921[degron::prg-1]) I; neSi82(Psun-1::tir-1::mRuby::sun-1 3'utr) II*	CRSIPR
EZS219	*glh-2(ne4922[flag::tev::degron::glh-2]) I; neSi82(Psun-1::tir-1::mRuby::sun-1 3'utr) II*	CRSIPR
EZS243	*glh-1(ne4926) glh-2(ne4922[flag::tev::degron::glh-2]) I; neSi82(Psun-1::tir-1::mRuby::sun-1 3'utr) II*	CRSIPR
EZS244	*glh-3(ne4923[flag::tev::degron::glh-3]) I; neSi82(Psun-1::tir-1::mRuby::sun-1 3'utr) II*	CRSIPR
EZS220	*glh-1(ne4926) glh-3(ne4923[flag::tev::degron::glh-3]) I; neSi82(Psun-1::tir-1::mRuby::sun-1 3'utr) II*	CRSIPR
EZS146	*glh-4(ne4924[flag::tev::degron::glh-4]) I; neSi82(Psun-1::tir-1::mRuby::sun-1 3'utr) II*	CRSIPR
EZS149	*glh-1(ne4926) glh-4(ne4924[flag::tev::degron::glh-4]) I; neSi82(Psun-1::tir-1::mRuby::sun-1 3'utr) II*	CRSIPR
WM806	*glh-1(ne4902[K391A]) I; wago-1(ne4585[flag::wago-1]) I;*	CRSIPR
WM827	*wago-1(ne4585[3Xflag::TEV::SNAP::WAGO-1]) glh-1(ne4809[V5::aid::glh-1]) I*	CRSIPR
WM828	*wago-1(ne4585[3Xflag::TEV::SNAP::WAGO-1]) glh-1(ne4925[V5::aid::glh-1, K391A]) I*	CRSIPR
WM808	*glh-1(ne4903[3Xflag::tev::glh-1]) I;*	CRSIPR
WM810	*glh-1(ne4904[3Xflag::tev::glh-1, K391A]) I;*	CRSIPR
WM817	*glh-1(ne4903[3Xflag::tev::glh-1]) prg-1(ne4906) I*	CRSIPR
WM818	*glh-1(ne4903[3Xflag::tev::glh-1]) I; rde-3(ne4907) III*	CRSIPR
WM809	*glh-4(ne4905[3Xflag::tev::glh-4]) I*	CRSIPR
WM819	*glh-1(ne4914[null]) glh-4(ne4905[3Xflag::tev::glh-4]) I;*	CRSIPR

Table S4, CRISPR Oligos, Related to STAR methods:

name	sequence	gene	usage
DS3_glh-1N_sgRNA	TTTTCTGCGAAAATGTCTGA	glh-1	*glh-1(null), GFP::GLH-1, FLAG::tev::glh-1*
DS10_glh-1C_sgRNA	TCCCTCAAGATGAAGAAGGC	glh-1	*glh-1(null)*
DS16_glh1K391A_sgRNA	GACGAGTCATGATAGGCAGA	glh-1	*glh-1 K391A*
DS19_glh1DQAD_sgRNA	TCAATTATGACAGTATGCCG	glh-1	*glh-1 DQAD*
DS27_glh1ZF_sgRNA	CCCTTGGCTTGGCTGGATTG	glh-1	*glh-1 Zinc finger deletion*
DS8_glh4N_sgRNA	tctgtatcgtatcaacgATG	glh-4	*glh-4 null, flag::tev::glh-4*
DS23_glh4C_sgRNA	tctgtatcgtatcaacgATG	glh-4	*glh-4 nul*
SD111_gfpglh1AID_sgRNA	ggaggtttggctggatcttt	glh-1	*gfp::degron::glh-1*
DS58_prg1N_sgRNA	taatatttttcaggtaataA	prg-1	*degron::prg-1, prg-1 mutant*
DS69_glh-2N_sgRNA	gttcgaagATGTCTGACGAT	glh-2	*Flag::tev::degron::glh-2*
DS85_glh-3N_sgRNA	agttaaacggcacggaataA	glh-3	*Flag::tev::degron::glh-3*
DS81_rde3N_sgRNA	GGACTTCGCTGATCTTTATT	rde-3	*rde-3 mutant*
name	sequence	gene	*usage*
DS11_glh-1null_donor	attttctggaaaaatcttaattttctgcgaaaATGTAGaaaaccgaccaattgatagtgtttcgcattta	glh-1	*glh-1 null*
DS18_glh-1K391A_donor	ATGATATCATGGCTTGTGCTCAAACTGGATCcGGA gct ACCGCTGCATTtCTTCTGCCTATCATGACTCGTCT	glh-1	*glh-1 K391A*
DS9_glh4_null_donor	ataatcaaacacgactctgtatcgtatcaacgATG CACCACCACCACCACCAC AGgtatccatttaacaaactagatcatttgcaaat	glh-4	*glh-4 null*

DS21_glh-1Nflag_donor	attttctggaaaaatcttaattttctgcgaaaATG GATTACAAAGACCATGATGGTGACTATAAGGATCATGATATTGACTATAA AGACGATGACGATAAG GAGAACCTCTACTTCCAATCG TCTGATGGTTGGAGTGATAGCGAAAGTGCTGCTAA	glh-1	*flag::tev::glh-1*
DS121_glh-1NV5_donor	attttctggaaaaatcttaattttctgcgaaaATG GGAAAGCCAATCCCAAACCCACTCCTCGGACTCGACTCTACC cctaaggacccggccaaacctccggccaaggcaca	glh-1	*V5::degron::glh-1*
DS107_prg1Ndegron_donor	attgttttctataatatttttcaggtaataATG cctaaagatccagccaaacctccggccaaggcacaagttgtgggatggccaccggtgagatcataccg gaagaacgtgatggtttcctgccaaaaatcaagcggtggcccggaggcggcggcgttcgtgaag GCATCTGGAAGTGGTCGCGGGAGAGGCCGTGG	prg-1	*Degron::prg-1*
DS_rde3indel_donor	ataataattgaatattttttgtagtaacaacaATG AAATAAAGATCAGCGAAGTCCAGAAGACATAGACT	rde-3	*rde-3 mutant*
